# Hendra and Nipah viruses: Biosafety evidence for risk-based containment, inactivation practices, and one health preparedness

**DOI:** 10.1016/j.onehlt.2026.101486

**Published:** 2026-06-19

**Authors:** S.D. Blacksell, K.K. Le, P.W. Selleck, J.R. Young, J.B. Kolenchery, J.T. Paulley, G.A. Marsh, M.P. Ward, L.J. Gleeson

**Affiliations:** a Mahidol Oxford Tropical Medicine Research Unit, Faculty of Tropical Medicine, Mahidol University, Bangkok, Thailand; b Centre for Tropical Medicine and Global Health, Nuffield Department of Medicine, University of Oxford, Oxford, United Kingdom; c Sydney School of Veterinary Science, The University of Sydney, Camperdown, NSW, Australia; d Australian Centre for Disease Preparedness, CSIRO, Geelong, VIC, Australia

**Keywords:** Hendra virus, Nipah virus, Biosafety, Biosecurity, Risk-based containment, Occupational exposure, Infection prevention and control, Laboratory biosafety

## Abstract

Hendra virus (HeV) and Nipah virus (NiV) are highly pathogenic zoonotic henipaviruses that pose persistent risks at the human–animal–environment interface. Implementation of the WHO Laboratory Biosafety Manual fourth edition (LBM4) requires evidence-based risk assessments tailored to specific pathogens, activities, and operational settings; however, the adequacy of biosafety evidence supporting risk-based containment decisions for henipaviruses remains unclear. Spillover from bat reservoirs, amplification in domestic animals, and subsequent human infection have resulted in recurrent outbreaks with high case fatality rates and significant occupational exposure risks for veterinarians, healthcare workers, and laboratory personnel. Effective management of these hazards requires coordinated biosafety approaches across human, animal, and laboratory systems within a One Health framework.

We conducted a structured narrative review of evidence relevant to biosafety risk assessment for HeV and NiV, focusing on laboratory diagnostics, occupational exposure, and validated inactivation and decontamination practices. Evidence from experimental studies, outbreak investigations, occupational exposure reports, regulatory guidance, and biosafety literature was critically synthesised across human, veterinary, and laboratory domains using the LBM4 risk-based framework as an organising structure.

Substantial gaps were identified in the biosafety evidence base. Human infectious dose thresholds remain undefined, transmission pathways, particularly for NiV, are incompletely characterised, and many recommended inactivation and decontamination procedures lack formal validation across relevant matrices and operational contexts. Key risk factors underpinning spillover and occupational exposure are poorly quantified, and for HeV, the causes of pronounced spatial and temporal clustering of cases remain unresolved. These limitations complicate risk-based decision-making, particularly for diagnostic, field, and laboratory activities conducted outside maximum containment facilities. Addressing these evidence gaps will strengthen implementation of the WHO LBM4 risk-based framework, improve protection of laboratory and field personnel, and enhance One Health preparedness for future spillover events.

## 1 Introduction

Hendra virus (HeV) and Nipah virus (NiV) are highly pathogenic zoonotic henipaviruses that exemplify the interconnected risks at the human-animal-environment interface. Spillover from bat reservoirs, amplification in domestic animals in some cases, and subsequent human infection have resulted in recurrent outbreaks with high case fatality rates and substantial occupational risk for veterinarians, healthcare workers, laboratory personnel, and others involved in animal and human health responses, particularly in settings where access to maximum containment facilities is limited [1]. These high case fatality rates, zoonotic transmission pathways, and occupational exposure risks place henipaviruses within a One Health framework, requiring coordinated approaches to prevention, diagnostics, biosafety, and outbreak preparedness across sectors.

Henipavirus emergence and spillover are inherently One Health problems because transmission occurs across interconnected human, animal, and environmental systems. In this review, One Health serves as the contextual framework for understanding how ecological change, wildlife reservoir dynamics, domestic animal amplification, healthcare-associated transmission, and laboratory activities contribute to exposure risk. However, the primary focus of the review is the biosafety evidence required to support risk assessment and implementation of laboratory biosafety measures.

The World Health Organization Laboratory Biosafety Manual (LBM4) [2], first published in 1983 and substantially revised in its fourth edition in 2020, introduced a major shift from prescriptive containment requirements based largely on pathogen classification toward a risk-based, activity-driven approach. Under this framework, biosafety measures are selected based on the hazards posed by specific activities, materials, procedures, and operational settings rather than solely on pathogen risk group classification. For high-consequence zoonotic pathogens such as HeV and NiV, the successful implementation of this approach depends critically on the availability and quality of evidence underpinning assumptions about infectious dose, transmission pathways, activities performed, and the effectiveness of inactivation and decontamination methods.

Despite the importance of HeV and NiV as high-consequence zoonotic pathogens, it remains unclear whether the available biosafety evidence is sufficient to support risk-based implementation of the WHO LBM4 framework. To date, no review has systematically examined the extent to which existing evidence supports implementation of the WHO LBM4 risk-based framework for henipaviruses. The aim of this review was therefore to critically evaluate the evidence relevant to biosafety risk assessment for HeV and NiV, identify strengths and limitations in the current evidence base, and highlight priority research needs to support laboratory worker safety and One Health preparedness.

Biosafety evidence relevant to henipaviruses spans experimental laboratory studies, outbreak investigations, occupational exposure reports, regulatory guidance, and operational biosafety practice documents across human, veterinary, and environmental domains. Given the heterogeneity of these evidence sources and their relevance to risk-based containment decision-making, a structured narrative review approach was used to critically synthesise current evidence, identify areas of strong and weak support for WHO LBM4 implementation, and highlight priorities for future research and preparedness.

## 2 Methods

### 2.1 Review approach

This study was undertaken as a structured narrative review examining biosafety evidence relevant to risk-based containment and laboratory management of HeV and NiV. The review was organised using the WHO LBM4 framework as a conceptual structure for evaluating evidence relevant to laboratory, diagnostic, occupational, and field biosafety practices.

### 2.2 Literature sources

Searches were conducted between November 2025 and February 2026. Evidence sources included peer-reviewed literature identified through PubMed/MEDLINE, Embase, and Web of Science, together with relevant guidance documents from the World Health Organization, CDC, national veterinary and public health agencies, biosafety manuals, outbreak investigation reports, and other relevant grey literature. No formal date restrictions were applied due to the relatively limited and episodic nature of henipavirus research.

### 2.3 Evidence selection and synthesis

Evidence was included if it addressed one or more biosafety-relevant domains, including transmission pathways, infectious dose, occupational exposure, diagnostic workflows, laboratory containment, inactivation and decontamination procedures, or biosafety implementation considerations relevant to HeV or NiV. Literature focused solely on molecular virology, pathogenesis, or vaccine development without clear biosafety implications was excluded.

Evidence was synthesised narratively and organised according to major biosafety domains relevant to WHO LBM4 implementation, including transmission risk, occupational exposure, diagnostics, inactivation procedures, containment considerations, and One Health prevention strategies. Emphasis was placed on identifying areas where biosafety practice is supported by experimentally validated evidence versus areas where precautionary assumptions or limited data currently guide decision-making. Where evidence applies similarly to both viruses, findings are presented together; where evidence differs substantially, HeV and NiV are discussed separately.

## 3 Results

Findings are organised according to key biosafety domains relevant to implementation of the WHO LBM4 framework, including pathogen characteristics, transmission pathways, infectious dose, occupational exposure, diagnostic workflows, inactivation and decontamination procedures, containment considerations, and One Health prevention strategies.

### 3.1 Current knowledge of HeV and NiV

#### 3.1.1 Characteristics of HeV and NiV

HeV and NiV are single-stranded RNA viruses from the Paramyxoviridae family, order Mononegavirales, and genus Henipavirus. Unlike other paramyxoviruses, Henipaviruses have a larger genome, approximately 15% longer, setting them apart within the family. NiV infects a wide range of hosts, causing severe illness and fatalities [3], with a case fatality rate of up to 75% in humans [4], [5].

### 3.2 Geographic distribution

#### 3.2.1 Hendra virus

HeV was first identified in 1994 in the suburb of Hendra in Brisbane, Queensland, Australia, following a fatal outbreak in horses and one human. Recent ecological and epidemiological evidence suggests that the geographic distribution and spillover dynamics of HeV may be shifting, including southward expansion associated with changes in bat ecology and the identification of novel HeV variants in regions previously considered lower risk [6], [7].

#### 3.2.2 Nipah virus

NiV emerged in 1998 during an outbreak among pigs and humans near Ipoh, Malaysia, referred to as strain NiV-Malaysia, with subsequent cases reported in Singapore in 1999 [8]. Subsequently, NiV human infections have been reported in Bangladesh (Dhaka, Rajshahi, Rangpur, Khulna, Chattogram, Mymensingh, and Barishal) referred to as strain NiV- Bangladesh, India (West Bengal and Kerala), and the Philippines (Sultan Kudarat) [9]. Since 2001, NiV infections in humans have been reported every year, except for 2006 [9], [10]. More recently, human NiV infections have also been reported in West Bengal, India, which borders Bangladesh [11]. Serological evidence of NiV has since been detected in multiple regions, including Vietnam [12], West Africa [13], China [14], Bangladesh, Thailand [15], and India [16]. The distribution of these two viruses is associated with that of *Pteropus* spp. fruit bats.

### 3.3 Clinical disease in humans

#### 3.3.1 Hendra virus

HeV infection in humans is a severe zoonotic disease with a reported case fatality rate of approximately 57% (4 of 7 recognised cases). Following an incubation period of between 7 and 16 days, patients typically develop an influenza-like illness that can rapidly progress to meningitis and/or encephalitis, often accompanied by seizures, neurological deterioration, and coma. Most fatal cases have resulted from severe encephalitis, although severe respiratory disease, multiorgan failure, and arterial thrombosis have also been reported [9], [17], [18].

#### 3.3.2 Nipah virus

NiV infection in humans typically presents as severe encephalitis, acute respiratory distress syndrome (ARDS), or a combination of neurological and respiratory disease. Clinical manifestations commonly include fever, headache, drowsiness, confusion, altered consciousness, vomiting, and respiratory symptoms such as cough, breathlessness, and hypoxia. Case fatality rates have varied between outbreaks, ranging from approximately 40% during the Malaysian outbreak to substantially higher levels in some outbreaks in Bangladesh and India. The incubation period is generally between 4 and 14 days, with a median of approximately 9 days. Although symptomatic disease is often severe and rapidly progressive, asymptomatic and subclinical infections have also been documented, particularly among close contacts and healthcare workers. Neurological complications, including encephalitis, intracerebral haemorrhage, and long-term brain lesions detectable by MRI, are characteristic features of severe infection [9], [19], [20], [21].

### 3.4 Pathogen transmission

#### 3.4.1 Hendra virus

Bats serve as natural reservoirs for HeV [22], [23] (See Table S1 for full details), including *Pteropus conspicillatus* [24], *P. scapulatus* [25], *P. alecto* [22], [26], and *P. poliocephalus* [22], [27]. HeV is primarily transmitted via bat urine [28], which can infect other bats, horses [29], and dogs [30]. Horses serve as dead-end hosts, and the virus is present in multiple organs, including the oral and nasal secretions. As of January 2026, there is no documented evidence of human-to-human HeV transmission [9], [31]. From a biosafety perspective, the aberrant host transmission pathways pose potential exposure risks during animal handling, necropsy, and processing of tissues and bodily fluids, underscoring the need for stringent controls even in non-laboratory settings.

#### 3.4.2 Nipah virus

Human NiV infections from consuming raw date palm sap [32], [33], [34], [35] contaminated with NiV-infected bat saliva and urine [36] have been documented in Bangladesh (See Table S2 for a summary). Video evidence has shown bats frequently visiting date palm trees and directly contaminating the sap. Studies demonstrate that a 10% increase in households consuming raw sap raises the odds of a NiV case within a village by a factor of 6.39 [37]. NiV transmission to humans could also occur through contact with infected livestock such as pigs, and domestic animals, such as cats, ferrets, and rodents [38], [39], [40], [41], underscoring the zoonotic nature of this pathogen. Evidence of patient-associated NiV infection via fomites has been reported [42], [43], particularly in hospital settings [9], [44], [45], [46], [47], emphasising the importance of increased biosafety measures during clinical sample handling and laboratory work with respiratory and bodily fluids, especially where aerosol formation and droplet or splash risks are present, to account for the potential infection route through ingestion. Human-to-human transmission of the NiV-Bangladesh strain is rapid and common in healthcare settings [9]. Person-to-person transmission was rare during the Malaysian NiV outbreak [9]. Epidemiological and modelling studies indicate that the basic reproduction number (R₀) of NiV in human disease is generally below 1, often estimated at around 0.5 in Bangladesh outbreak analyses [48], reflecting limited sustained human-to-human transmission despite high case fatality rates.

### 3.5 Infectious dose

Human infectious dose for HeV and NiV infections remains unknown and cannot be inferred directly from animal aerosol or intranasal challenge models. However, studies in animal models provide some insights (See Tables S1 & S2 for a summary). In the absence of defined human infectious dose thresholds, biosafety risk assessments should adopt a precautionary approach, assuming the potential for infection at low doses, including via aerosol exposure, and apply conservative controls across all plausible exposure routes when handling live virus or potentially infectious material.

#### 3.5.1 Hendra virus

Guinea pigs were susceptible to HeV doses as low as 50 TCID_50_ [49], with high doses (30,000–50,000 TCID_50_) causing neurological lesions [50].

#### 3.5.2 Nipah virus

In African Green Monkeys, NiV lethality occurred 9–12 days post-infection with doses ranging from ∼2.5 × 10^3^ to ∼1.3 × 10^6^ plaque-forming units (PFU) [51]. Syrian hamsters developed respiratory or neurological disease after receiving 10^7^ 50% Tissue Culture Infectious Doses (TCID_50_) via oesophageal injection or ingesting synthetic palm sap [52]. The Malaysian prototype NiV strain (199901924) exhibited a 50% mean lethal dose of fewer than 10^2^ PFU per animal via aerosol in Syrian Hamsters [53]. In cats, clinical symptoms such as fever and dermal hemorrhagic lesions appeared after inoculation with 500 TCID_50_ of NiV [54]. Ferrets developed disease closely mirroring human infections at similar doses [55].

#### 3.5.3 Disinfection and decontamination

A summary of HeV and NiV decontamination and inactivation methods is presented in Table 1; full details are provided in Table S3. Evidence for henipavirus inactivation varies substantially across methods, matrices, virus titre and experimental conditions. Validated inactivation data are matrix-specific and largely derived from NiV rather than HeV. Where inactivation methods have not been formally validated for specific matrices or operational conditions, laboratories should avoid assuming equivalence. They should treat materials as potentially infectious until inactivation efficacy has been demonstrated. In low-resource diagnostic settings, chemical disinfectants are often the most practical and widely applicable means of inactivating HeV and NiV; however, their selection, efficacy, and proper application must be well understood to ensure biosafety and prevent inadvertent exposure.

**Table 1:** Summary of experimentally validated chemical, physical, and thermal disinfection and inactivation methods for Hendra virus (HeV) and Nipah virus (NiV). Conditions, matrices, and contact times vary substantially between studies; inactivation efficacy should not be assumed without validation for the specific specimen type and operational context. A value of ‘Yes’ under ‘Generalisable across matrices/settings’ indicates evidence considered broadly applicable beyond the specific experimental conditions tested, whereas ‘No’ indicates validation limited to the tested conditions only.

Virus	Method category	Disinfectant / treatment	Conditions (concentration, time, matrix)	Outcome	Reference	Generalisable across matrices/settings
HeV	Chemical	Paraformaldehyde (PFA)	4% PFA, 15 min; infected cell monolayers	Complete inactivation; >8-log reduction in infectious titre	[56]	Yes
	Radiation	Gamma irradiation	50 kGy; cell culture material	Complete inactivation confirmed by infectivity assay and serial passage	[64], [65]	Yes
NiV	Chemical	Neutral buffered formalin (NBF)	10% NBF; biological samples	Complete inactivation	[56]	No
		Formalin	0.1% for 72 h at 4°C (cells); 10% for 24 h (culture medium)	Complete inactivation	[59], [61]	No
		Sodium hypochlorite	10%; suspension tests	Infectious virus reduced to undetectable levels	[59]	No
		Ethanol	80% (rapid); 19% for ≥8 min	Complete inactivation	[59], [60]	No
		Detergents / extraction reagents	SDS, Triton X-100, AVL, RLT, TRIzol; cells/supernatant	No detectable infectious virus or viral RNA after passage	[62]	No
		Organic solvents	Acetone, methanol	Incomplete or variable inactivation; residual RNA detected	[62]	No
		Commercial disinfectants	Micro-Chem Plus, FWD, medical ethanol; 15 s	>4-log reduction in infectivity	[60]	No
		Binary ethylenimine (BEI)	6 h (confirmed by titration); 24 h safety testing	Complete inactivation	[63]	No
	Radiation	Gamma irradiation	50 kGy; culture material	Complete inactivation confirmed by infectivity assay	[64], [65]	No
	UV light	UV-C (254 nm)	1 h exposure in supernatant	Complete inactivation	[62]	No
		UV-C (254 nm)	10 min insufficient in serum; 30 min effective	Matrix-dependent inactivation	[66]	No
	Chemical + light	UVC	0.2 J/cm^2^; platelet concentrates	≥4-log reduction to LOD	[67]	No
	Chemical + light	Methylene blue + visible light	10 μM MB + 50,000 lx for 30 min	Complete inactivation	[68]	No
	Heat	Heat treatment	56 °C for 30 min: ≥4-log reduction; occasional residual infectivity	Partial inactivation	[66]	No
		Heat treatment	56 °C for 60 min or 60 °C for 30 min	Complete inactivation in all replicates	[66]	No

HeV – Hendra virus; NiV – Nipah virus; PFA – Paraformaldehyde; NBF – Neutral buffered formalin; BEI – Binary ethylenimine; SDS – Sodium dodecyl sulfate; AVL – Guanidinium thiocyanate–containing lysis buffer (Qiagen); RLT – Guanidinium thiocyanate–containing lysis buffer (Qiagen); TRIzol – Phenol–guanidinium thiocyanate reagent for nucleic acid extraction; UV / UV-C – Ultraviolet light / ultraviolet C (≈254 nm wavelength); UVC – Ultraviolet C irradiation; MB – Methylene blue; kGy – Kilogray (unit of absorbed ionising radiation dose); TCID₅₀ – 50% tissue culture infectious dose; LOD – Limit of detection.

### 3.6 Chemical disinfectants

#### 3.6.1 Hendra virus

Research on laboratory inactivation of HeV is limited. Complete inactivation of HeV in Vero cells has been achieved with 4% paraformaldehyde for 15 min [56]. The Australian Government recommends disinfectants for human and fomite decontamination, including soap, detergent, 2% glutaraldehyde, 10% formalin, hypochlorite, and commercial products (Virkon®S, Micro-Chem Plus) [57]. However, outbreak reports and operational guidance, such as AUSVETPLAN [57], rely on laboratory data and precautionary principles. In most instances, quantitative validation of disinfectant performance in field outbreak settings is limited or lacking, so recommendations are primarily evidence-informed rather than outbreak-validated.

### 3.7 Nipah virus

NiV is highly stable in blood stored in sealed containers at ambient temperature for up to 7 days [58], highlighting the need for effective virus-inactivation protocols and complete personal protective equipment (PPE) when handling blood samples. For NiV, effective inactivation to undetectable levels has been demonstrated using 10% neutral buffered formalin [56], 10% sodium hypochlorite [59], 38–80% ethanol [59], [60], and 0.1–10% formalin [61]. Although NiV RNA can still be detected after treatment with organic solvents like acetone and methanol, other reagents, including SDS (10 min, 45 °C), Triton-X 100 (20 min, room temperature/56 °C), AVL, RLT, and TRIzol (20 min, room temperature), have shown efficiency in eliminating detectable virus genomes in various sample types (cell, supernatant, and organ) [62]. Three disinfectants, Micro-Chem Plus, FWD (Forward DC, Diversey®, quaternary ammonium-based), and medical ethanol (38–76%), achieved >4 log NiV inactivation within 15 s [60]. Complete inactivation of NiV has also been achieved using BEI (1.025 g 2-bromoethylamine and 0.2 N NaOH at 3 mM) at room temperature for 24 h, allowing treated samples to be safely handled in BSL-2 facilities [63].

#### 3.7.1 Radiation

Henipaviruses can be effectively inactivated through radiation-based methods. Complete inactivation was achieved using four doses (three cycles per dose) from a cobalt-60 source with a JL Shepherd Model 484R irradiator [64] or 50 kGy gamma irradiation [65]. NiV in serum samples can also be inactivated by UV light within 1 h at room temperature [62] or by UV irradiation (312 nm wavelength, 2.5 mW/cm^2^) with an aluminium foil overlay for 10–30 min [66]. No equivalent radiation-based inactivation studies were identified for HeV.

#### 3.7.2 Combinations of chemicals and light

Combinations of chemicals and UV are effective for inactivating Henipavirus. THERAFLEX UV Platelets (UVC) and THERAFLEX MB Plasma (methylene blue/light) are effective in reducing the infection efficiency of NiV: the UVC dose of 0.02 J/cm^2^ in platelet concentrates reduces the infection efficiency by three-quarters, and a dose of 120 J/cm^2^ in plasma reduces it by one-quarter [67]. In addition, NiV on the surface of N95 masks (3 M 1860) and KN95 masks (Chengde Technology CD9501B) was completely inactivated by combining 10 μM methylene blue with intense light (50,000 lx) [68]. However, these methods are less likely to be amenable in low-resource settings.

#### 3.7.3 Heat

Heat is an effective method for inactivating the virus. By heating plasma samples at 56 °C for 60 min and 60 °C for 30 min, NiV was inactivated to undetectable levels, whereas heating at 56 °C for 30 min can still detect residual virus [66].

### 3.8 Challenges in biosafety management

#### 3.8.1 Risk group classification and biocontainment considerations

HeV and NiV are classified as Risk Group 4 (RG4) pathogens. However, under the WHO's risk-based approach outlined in the LBM4 [69], biosafety levels are determined by the activity performed (e.g., PCR, in vitro growth, or in vivo experiments) rather than the pathogen's RG classification (i.e., RG does not equal BSL) [69]. This approach emphasises risk assessment to dictate containment and work practices, avoiding the impracticality of defaulting to biosafety level 4 (BSL-4) for all activities [70], [71]. Advancements such as NiV [72], [73], [74], [75] and HeV [75] pseudoviruses, as well as other synthetic systems, enable functional assays to be safely conducted under BSL-2 conditions, providing practical and sustainable alternatives for specific research or diagnostic activities. The nature of the material should guide risk-based containment decisions for henipaviruses, including the potential for aerosol generation and the availability of validated inactivation procedures, rather than solely by Risk Group classification. In addition, these assessments should account for the exposure setting, including the number of individuals who could be affected and the degree of environmental control, with higher-occupancy or less controlled settings warranting more conservative containment and procedural safeguards.

### 3.9 Biosecurity management

In the United States, HeV and NiV are classified as Select Agents under the Code of Federal Regulations (7 CFR Part 331, 9 CFR Part 121, and 42 CFR Part 73). This classification imposes strict regulations on the possession, storage, use, and transport of these pathogens [76], [77]. As of January 16, 2025, NiV is designated as a Tier 1 select agent under the U.S. Federal Select Agent Program, reflecting its potential to pose a severe threat to public and animal health due to high case fatality, person-to-person transmission, and the lack of widely accessible countermeasures. [78].

### 3.10 Occupationally acquired infections

#### 3.10.1 Hendra virus

To date, all human HeV cases have resulted from close contact with infected horses during procedures like postmortem examinations or endoscopies; seven confirmed cases of HeV infection have resulted from significant exposure to horse body fluids [79], with four fatalities (Table S1). These included two Australian practising veterinarians in 2008 and 2009 and a racehorse trainer in 1994 [80].

#### 3.10.2 Nipah virus

During the 1998–1999 NiV outbreak in Malaysia, virus infection occurred in military personnel involved in culling operations [81] and workers in direct contact with live swine or fresh pork products [82]. Similarly, in the 1999 Singapore outbreak, which occurred in pigs transported from the outbreak area, all reported infections occurred among predominantly male slaughterhouse workers employed during the outbreak period [83]. Human-to-human transmission of NiV has been demonstrated in hospital settings [46], [47], [84]. The virus has been detected in the respiratory secretions and urine of infected individuals, suggesting transmission via contact with contaminated body fluids [85], [86]. Outbreaks in India (Siliguri, West Bengal, in 2001; Kerala, in 2018) predominantly show nosocomial spread, with healthcare workers and hospital contacts accounting for most cases, and transmission occurring across wards, emergency rooms, and radiology areas [9]. However, hospital laboratory workers were exposed to NiV during outbreaks in Malaysia (1998–1999) [45] and Bangladesh (2004, 2013–2014) [44], [87] but did not become infected.

Despite these risks, no documented laboratory-acquired HeV or NiV infections have been reported to date, likely reflecting limited global handling of live virus under high-containment conditions. The absence of documented laboratory-acquired henipavirus infections likely reflects robust microbiological laboratory safety standards and practices rather than low intrinsic risk and should not be interpreted as evidence that reduced biosafety controls are safe.

### 3.11 HeV and NiV diagnostics and implications for biosafety

#### 3.11.1 Hendra virus

**Fig. 1:**
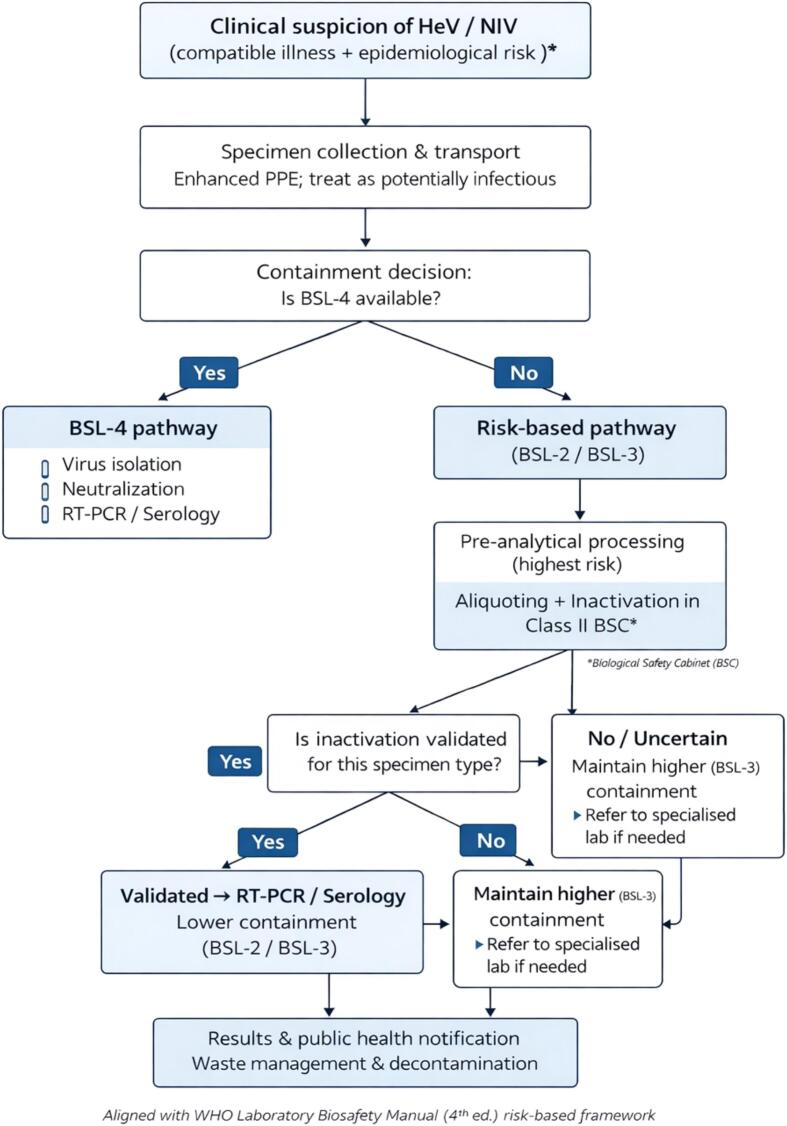
Schematic overview of a risk-based diagnostic workflow for suspected Hendra virus (HeV) and Nipah virus (NiV) infection. The highest biosafety risk occurs during specimen collection and pre-analytical processing prior to validated inactivation. Decision points reflect the availability of maximum containment and uncertainty in inactivation efficacy, consistent with the WHO Laboratory Biosafety Manual (4th edition) principles.

Laboratory diagnosis of Hendra virus (HeV) infection relies primarily on the detection of viral RNA by real-time reverse transcription PCR (RT-PCR) in clinical specimens, including blood, respiratory secretions, urine, cerebrospinal fluid, and tissues collected from humans or animals [31], [65]. Serological methods, including IgM and IgG enzyme-linked immunosorbent assays (ELISAs) and virus neutralisation tests, are used to support diagnosis, retrospective investigations, and surveillance activities. Virus isolation and neutralisation assays remain reference methods for confirming infection; however, these procedures require Biosafety Level 4 (BSL-4) containment and are therefore restricted to specialised high-containment facilities. Biosafety considerations for HeV diagnostics are particularly important during specimen collection, packaging, transport, processing, and inactivation, where potentially infectious materials may be handled before diagnosis is confirmed. Consequently, risk-based diagnostic workflows should ensure that containment measures, validated inactivation procedures, and laboratory practices are aligned with the nature of the specimen and the activities being performed [9], [31], [69]. Uncertainty surrounding the human infectious dose and potential transmission routes further complicates risk assessment for diagnostic laboratories, underscoring the need for structured, risk-based diagnostic workflows that align testing methods with validated inactivation procedures and appropriate containment levels during HeV and NiV outbreaks (Fig. 1). A summary of suggested biosafety approaches for diagnostic activities is presented in Table S2.

#### 3.11.2 Nipah virus

In outbreak settings, initial diagnostic testing is frequently performed in laboratories operating at BSL-2 or BSL-3 using molecular assays following chemical or heat inactivation of specimens [56], [62], [66]. The highest biosafety risk occurs during the pre-analytical phase, encompassing specimen collection, packaging, transport, aliquoting, and inactivation, when viral loads may be high and infection status unknown [44], [45], [46], [47], [87]. Although several inactivation reagents (e.g., guanidinium-based buffers, detergents, formalin, and heat) are widely used prior to molecular testing, validation across specimen types and operational conditions is incomplete, and residual infectivity cannot always be excluded [56], [62].

### 3.12 Review of existing biosafety measures

#### 3.12.1 HeV and NiV risk assessments and biosafety management

Laboratory personnel, veterinarians and slaughterhouse workers face a high risk of contracting HeV and NiV if they lack appropriate PPE and have direct contact with infected sources. No HeV cases have been reported among individuals documented as using appropriate PPE during exposure events [88]. A summary of biosafety and biocontainment requirements for handling HeV and NiV, emphasising strict protocols to mitigate risks in high-risk procedures such as clinical sample collection, transport, processing, serological testing, and virus isolation, is presented in Table 2. Personnel risks, including aerosol and splash exposure and needlestick injuries, are mitigated through primary and secondary containment measures. Primary containment is provided by the use of appropriate PPE (e.g., N95 respirators or powered air-purifying respirators), strict adherence to good microbiological practices, and physical containment devices, including biological safety cabinets (BSCs) or a BSL-4 cabinet line (i.e., negative-pressure Class III BSCs), which directly protect personnel from aerosol and contact exposure. Activities involving high viral loads are conducted under BSL-4 conditions, with secondary containment provided by the laboratory itself, including controlled access, directional airflow, sealed surfaces, and validated decontamination systems. In experimental animal work, BSCs are not used because the BSL-4 laboratory space functions as the secondary containment barrier, while PPE remains the primary means of personnel protection.

**Table 2:** Detailed risk assessment and control mitigations for working with Hendra (HeV) and Nipah (NiV) viruses.

Procedure	Sample type	Hazard	Initial risk	Risk mitigation	Residual risk (Assuming validated controls and compliance)	Reference
Examination of a sick horse (clinical exam, nasopharyngeal/oral swab collection, blood sampling, rectal/urine sampling)	Oral/nasal swab in VTM or lysis buffer; blood; urine; body fluids	− Aerosol/splash exposure from respiratory secretions; close contact with infected animal; needlestick/sharp injury; contamination of clothing/equipment	Very High	− Treat as suspected HeV/NiV case: enhanced PPE (impervious coverall/gown, double gloves, eye protection/face shield). − Respiratory protection: fit-tested N95/P2 or PAPR where available/appropriate (PAPR recommended for prolonged close contact or when aerosol generation likely). − Minimise personnel in contact (one experienced clinician only). Keep animal restrained to limit aerosol generation. − Prefer sampling that minimises aerosol generation; use nasopharyngeal/oral swabs placed directly into validated inactivating buffer where possible. If VTM is used, treat as infectious and avoid open handling. − Use sealed transport containers and triple packaging; label as high-risk and notify receiving lab. − Ensure immediate on-site decontamination of surfaces and equipment (recommended disinfectants listed in Table 4: 10% bleach, 70–80% ethanol, formalin/paraformaldehyde as appropriate). − Document emergency response & exposure reporting procedures; ensure staff trained and current in PPE donning/doffing.	Low	[29], [88], [91], [93]
Clinical or non-clinical sample collection	Oral or Nasal Swab in VTM or lysis buffer Tissue Blood Serum	− Aerosol or splash exposure during sample collection, needlestick or injury from contaminated sharp (Clinical collection or necropsy) − Needlestick injuries	High	− Standard PPE⁎ − N95 Respirator or powered air purifying respirator (PAPR) ⁎⁎ − GMPP⁎⁎⁎ − Validated waste management for infectious materials † − Standard disinfection and decontamination†† − Emergency response procedures and associated staff training †††	Low	[10], [31], [56], [123]
Sample transport	Oral or Nasal Swab in VTM or lysis buffer Tissue Blood Serum	− Leaking sample causing aerosol or splash exposure	High	− Samples should be packaged in triple layer packing: o water-proof primary container that contain samples and an absorbent material o water-proof secondary packaging o an outer packaging of adequate strength − Ensure staff are appropriately trained in IATA dangerous goods regulations and transport requirements − Emergency response procedures and associated staff training †††	Low	[10], [31], [56], [88], [123]
Serology (ELISA)	Serum	− Aerosol exposure during sample processing − Eye splash during sample processing	High	− As per *Sample collection* Note: N95 Respirator only if risk assessment indicates − Serology samples processed in certified Class II BSC‡‡ with risk assessment Note: Work in certified Class III BSC‡ or cabinet line only if risk assessment indicates when processing serum samples − Centrifugation using sealed centrifuge cups or rotors − Heat inactivation of serum at 56 °C for 60mins − After inactivation standard PPE	Low	[10], [31], [123]
Sample reception and/or sample processing	Oral or Nasal Swab in VTM or lysis buffer Tissue Blood Serum	− Leaking sample − Aerosol exposure during sample processing − Eye splash during sample processing	High	− Working under BSL-4 (maximum containment measures) biocontainment, including associated practices and procedures − PPE would normally require the use of positive pressure suits and decontamination procedures at the completion of laboratory activities − Work in certified Class III BSC‡ or cabinet line − Centrifugation using sealed centrifuge cups or rotors − GMPP⁎⁎⁎ − Validated waste management for infectious materials † − Standard disinfection and decontamination†† − Emergency response procedures and associated staff training ††† − PCR and serology samples can be processed in certified Class II BSC‡‡ with risk assessment and appropriate PPE	Low	[10], [31], [56], [62], [66], [123]
Nucleic acid amplification test (NAAT)	Oral or Nasal Swab in VTM or lysis buffer Tissue Blood	− Aerosol exposure during sample processing − Eye splash during sample processing − Infectious culture material spill	High	− As per *Sample reception/processing* for nucleic acid extraction only − Addition of extraction buffer must be in sample processing and extraction step location dependent on risk assessment and on inactivation of sample by extraction buffer − After extraction standard PPE	Low	[10], [31], [56], [62], [66]
Virus isolation	Oral or Nasal Swab in VTM or lysis buffer Tissue Blood	− Aerosol exposure during sample processing − Eye splash during sample processing − Infectious culture material spill − High virus concentration and volume	Very high	− Working under BSL-4 (Maximum containment measures) biocontainment, including associated practices and procedures − PPE would normally require the use of positive pressure suits and decontamination procedures at the completion of laboratory activities − Work in certified Class III BSC‡ or cabinet line − Centrifugation using sealed centrifuge cups or rotors − GMPP⁎⁎⁎ − Validated waste management for infectious materials† − Standard disinfection and decontamination†† − Emergency response procedures and associated staff training practiced †††	Low	[10], [31], [123]
Serology (Virus neutralisation)	Serum Concentrated live virus	− Aerosol exposure during sample processing − Eye splash during sample processing − Infectious culture material spill − High virus concentration and volume	Very high	− As per *Virus isolation*	Low	[10], [31], [57], [72], [73], [74], [75], [123]
Waste Disposal & Incomplete decontamination	Samples Consumables Waste	− Aerosol exposure during handling − Eye splash during sample handling − Contamination of environment	High	− Validated waste management for infectious materials† − Standard disinfection and decontamination†† − Standard PPE⁎ − N95 Respirator⁎⁎ − GMPP⁎⁎⁎ − Emergency response procedures and associated staff training †††	Low	[10], [57], [58], [59], [62], [87], [123]
Whole-genome sequencing	Nil	− Nil	Nil	− Not required	Nil	[10], [31], [62], [65], [123]
Exposure to chemicals	Not applicable	− Chemicals used for nucleic acid isolation or disinfection	Low	− **Consult material safety data sheets for each chemical prior to commencing work** ‡‡	Low	

⁎ Standard PPE - Lab coat or gown (or coverall as indicated by risk), gloves, eye protection or face shield, including documented training and competency in donning and doffing.

⁎⁎ N95 Respirator - fit tested before using it for the first time and perform fit testing annually.

⁎⁎⁎ GMPP - Good Microbiological Practices & Procedures (i.e., confirm staff competency).

† Validated waste management - best practice sharps and infectious biologicals disposal.

†† Recommended decontamination methods for HeV and NiV include 4% paraformaldehyde, 10% formalin, 10% sodium hypochlorite, or 70–80% ethanol for surfaces and equipment. Heat treatment at 56 °C for 60 min or 60 °C for 30 min is suitable for biological samples. Combined approaches, such as methylene blue with intense light, are effective for PPE decontamination. Steam sterilisation at 121 °C for 30 min. Note that all disinfection or sterilisation processes must be validated against the pathogen in question. Autoclave cycles must be regularly validated for complete sterilisation.

††† Emergency response procedures - including documented training and competency.

‡ Operate within an annually certified Class II/III BSC. Staff must receive training on the operation, maintenance, and use of BSCs.

‡‡ Identify hazards and implement risk mitigation strategies. Ensure that staff are trained in the safe use of chemicals, disposal, and emergency situations.

Strict decontamination protocols, including autoclaving, chemical showers upon personnel exit and dunk tanks for materials movement, ensure containment. At the same time, emergency response plans for spill management and post-exposure medical treatment are rigorously maintained. Antiviral agents such as Ribavirin may also be available for emergency use. This comprehensive approach, supported by extensive staff training and regular mock drills, ensures the highest level of safety when manipulating these highly pathogenic agents.

### 3.13 Prevention and One Health approach

#### 3.13.1 Prevention

##### 3.13.1.1 One Health approach in endemic areas

Implementing policies for epidemic preparedness and prevention is critical for high-consequence zoonotic RG4 agents such as HeV and NiV, which have demonstrated the ability to cause significant impacts to public health through recurring outbreaks, such as in Bangladesh from 2001 to 2014 [89].

#### 3.13.2 Hendra virus

In Australia, HeV prevention guidelines from the governments of Queensland [88], [90], [91] and New South Wales [92] emphasise biosecurity measures, informed by seasonal, environmental, and management-related risk factors, such as distancing horses from bat roosting and feeding sites, modifying paddock use during higher-risk periods, stabling horses when appropriate, and utilising the equine vaccine as the most effective preventive measure [93]. For HeV, the integrated One Health prevention strategies underscore the importance of equine vaccination campaigns [94]. However, challenges such as cost, safety concerns, and limited awareness among horse owners hinder widespread adoption [95], [96], [97]. Vaccinated horses develop antibodies against HeV, which may complicate international movement unless a DIVA (Differentiating Infected from Vaccinated Animals) system is developed. Supplementary ecological strategies have also been proposed, such as habitat management and bat deterrence, which are under consideration in Australia [93] and Singapore [98]. For HeV prevention, when broader social, ecological, animal welfare, and community impacts are included, vaccinating horses is always the preferred option [93], [99]. Bat roost removal is not a universally attractive economic option. However, a barrier to implementing such policy approaches is how to include broader ecological, social, economic, and animal welfare impacts in analyses and how to value them across the range of stakeholders who should be consulted [93].

#### 3.13.3 Nipah virus

Guidelines for NiV prevention are provided by India [100], [101] and the WHO [102], emphasising the importance of the One Health strategy for global health security [103], [104], [105], [106]. This approach integrates the management of humans, animals, and the environment to mitigate risks. Low-cost interventions, such as targeted posters and public service announcements, are effective strategies for raising awareness in populations vulnerable to NiV transmission [107]. To reduce sporadic NiV transmission, community interventions in sap production areas, such as the use of effective sap covers, should be promoted [39], [108]. These measures can prevent bat-sap contact and curb human infections, supporting broader epidemic prevention efforts. Bat roost disturbance may also have unintended ecological and One Health consequences, including stress-associated changes in bat movement and viral shedding dynamics.

#### 3.13.4 Vaccination

The only vaccination available for equines against HeV is Equivac® HeV, introduced in November 2012. It can produce protective antibody titres [109] and is officially approved by the Australian Pesticides and Veterinary Medicines Authority (APVMA) as a preventive equine vaccine [110]. No licensed human vaccine currently exists for either HeV or NiV infection, although several candidate vaccines remain under development.

#### 3.13.5 Human postexposure prophylaxis and treatment

No approved vaccines or postexposure therapies exist to prevent or treat human HeV and NiV infections. The human monoclonal antibody m102.4, which targets the viral glycoprotein G [111], has shown promise in animal models and has been used compassionately in humans; however, outcomes have been variable [79], and no formal human efficacy estimate is available. A phase 1 clinical trial demonstrated favourable safety, tolerability, pharmacokinetic, and immunogenicity profiles in healthy adults [112]. While ribavirin and chloroquine have demonstrated in vitro efficacy against HeV and NiV, their therapeutic use remains uncertain [113], [114]. Ribavirin may reduce mortality in acute NiV encephalitis, but the rate in treatment groups remains 32% [115]. In experimental studies, promising results have been observed with Remdesivir (GS-5734), which protected African green monkeys from NiV infection [116].

## 4 Discussion

### 4.1 Strengths and limitations of the current biosafety evidence base

Despite significant advances in understanding HeV and NiV biology and epidemiology, substantial gaps persist in the evidence on effective biosafety measures, particularly in laboratory and occupational settings. These gaps challenge the development of effective risk mitigation strategies and the safe work practices in situations of likely exposure to these RG4 pathogens. Collectively, these findings demonstrate that implementation of the WHO LBM4 risk-based framework for henipaviruses is constrained less by a lack of biosafety guidance than by gaps in the underlying evidence needed to support risk assessment decisions.

A critical gap in understanding HeV and NiV lies in the limited knowledge of the minimum infectious dose, particularly in humans. While animal studies provide valuable data on pathogenicity and dose-response relationships, they cannot fully account for the complexities of human exposure scenarios. This lack of knowledge impedes the development of tailored biosafety protocols and undermines efforts to establish risk-based PPE standards for high-risk groups, such as veterinarians and laboratory workers. While slaughterhouse personnel are at risk during unrecognised outbreaks of NiV, ongoing PPE use in such settings is often impractical; therefore, once a NiV outbreak in pigs is recognised, all animal movement from the affected area should be halted. Qualitative research involving veterinarians and horse owners has also highlighted operational difficulties associated with prolonged PPE use during equine management, including heat stress, communication barriers, and altered horse behaviour responses [117]. Historical NiV outbreaks emphasise the dangers of inadequate infection prevention measures. For instance, although dealing with an unknown agent, exposure during culling operations, direct contact with infected animals, or handling contaminated materials have significantly contributed to viral transmission. In these cases, the absence or misuse of PPE increased infection risk, underscoring the importance of more clearly defined dose thresholds and standardised risk mitigation measures to inform protective practices. Addressing this evidence gap is essential to mitigate potential outbreaks and ensure individual safety in both laboratory and field settings [81], [82], [83], [118]. Together, these observations indicate that while current evidence supports precautionary biosafety measures, substantial uncertainty remains regarding the relationship between exposure dose, transmission route, and occupational infection risk.

Inactivation protocols for HeV and NiV remain insufficiently validated, creating significant challenges for ensuring safe handling under diverse laboratory conditions. While laboratory studies demonstrate the efficacy of various chemical disinfectants, radiation-based methods, and heat treatments, their practicality and availability, particularly in lower containment settings such as BSL-2, require further investigation. For NiV, extensive research has evaluated inactivation methods for virus-infected cells, supernatants, and tissues, with one study examining 19 standard protocols [62]. However, data on the efficacy of these methods for environmental matrices or solid surfaces remain sparse. By comparison, HeV inactivation has been studied far less extensively, creating a critical imbalance despite both viruses being classified as high-risk henipaviruses. The limited availability of validated inactivation protocols from BSL-4 facilities underscores the need for robust and reliable references. Further evidence is essential to determine whether inactivated HeV and NiV samples can be safely handled in BSL-2 laboratories without unnecessary risks. Addressing these gaps would strengthen biosafety standards and support practical, scalable diagnostic and surveillance efforts, especially in resource-constrained settings. These limitations are particularly important for laboratories operating outside maximum containment facilities where validated inactivation procedures are central to safe diagnostic workflows.

Another critical gap is the inconsistency in biosafety regulations across regions. While the WHO's risk-based approach in the LBM4 provides a flexible framework for laboratory safety, its implementation varies widely. Laboratories in resource-limited settings may lack access to advanced containment facilities, such as BSL-4, leading to reliance on BSL-2 or BSL-3 conditions for inactivated samples. Collectively, the current biosafety evidence base for henipaviruses is characterised by substantial heterogeneity in evidence quality, operational applicability, and experimental validation. While important advances have been made in understanding transmission dynamics, occupational exposure risk, and laboratory inactivation procedures, many current biosafety practices still rely partly on precautionary assumptions because matrix-specific validation data remain incomplete across operational settings. Importantly, these evidence gaps do not imply that current biosafety practices are ineffective; rather, they highlight areas where stronger empirical evidence would increase confidence in risk-based decision-making under the WHO LBM4 framework.

### 4.2 Priority research needs

Although numerous evidence gaps were identified, several emerge as particularly important for the implementation of risk-based biosafety approaches. First, defining the infectious dose for HeV and NiV would substantially improve occupational risk assessment. Second, matrix-specific validation of inactivation and decontamination procedures is required to support safe diagnostic workflows outside maximum containment laboratories. Third, better quantification of occupational exposure pathways would strengthen evidence-based PPE recommendations. Fourth, implementation research involving laboratory personnel, veterinarians, healthcare workers, and biosafety practitioners is needed to understand how LBM4 guidance is interpreted and applied in practice. These priority research areas are summarised in Table 3. Finally, integration of One Health surveillance information into laboratory risk assessment frameworks may improve preparedness during periods of increased spillover risk.

**Table 3:** Priority biosafety research needs relevant to implementation of risk-based containment approaches for Hendra and Nipah viruses.

Evidence gap	Operational consequence	Suggested research priority
Human infectious dose	PPE uncertainty	Human exposure modelling
Matrix-specific inactivation	Diagnostic workflow uncertainty	Validation studies
Aerosol transmission	Containment uncertainty	Aerosol challenge studies
Environmental persistence	Spillover uncertainty	Environmental persistence studies
PPE implementation barriers	Occupational risk	Qualitative implementation studies

### 4.3 Implications for WHO LBM4 implementation

The principal contribution of this review is to evaluate whether the evidence currently available is sufficient to support implementation of the WHO LBM4 risk-based framework for henipaviruses. These evidence limitations have important implications for the implementation of the WHO LBM4 risk-based framework. Because the WHO LBM4 emphasises activity-based risk assessment rather than prescriptive pathogen classification alone, uncertainty regarding infectious dose, transmission pathways, aerosol-generation risk, and matrix-specific inactivation efficacy may necessitate more conservative containment decisions than would otherwise be required with stronger evidence.

However, the absence of validated inactivation procedures that reflect the specific circumstances of the work, combined with regional differences in laboratory practices, environmental conditions, and behavioural factors, remains underexplored and may increase the risk of accidental exposure [119], [120].

Although ecological drivers such as the marked increase in fruit-bat presence following deforestation have been identified as key upstream determinants of spillover [121], routine laboratory biosafety risk assessments rarely incorporate such landscape-level or seasonal signals; as a result, assessments should be adapted to include One Health surveillance triggers (for example increased local bat activity or seasonal peaks) so that containment and operational controls can be escalated during documented high-risk periods. These implementation challenges are likely to be most significant in lower-resource laboratory and field settings where sustainable and proportionate biosafety approaches depend heavily on confidence in validated inactivation procedures and locally appropriate operational risk assessments.

### 4.4 Implications for One Health preparedness

Transmission pathways, especially from bats to intermediary hosts, are also not fully understood, although fomite contamination with bat excreta is one likely route. The significant rise in fruit bat presence in Southeast Asian plantations between 1997 and 1998 was driven by extensive deforestation for pulpwood and the expansion of industrial crops over the preceding two decades [121]. Fruit bats, particularly *pteropids*, are natural hosts of HeV and NiV. Additionally, *Ixodes holocyclus* ticks, which parasitise flying foxes, have been proposed as a hypothetical link in henipavirus transmission; however, this hypothesis remains unsupported by experimental or epidemiological evidence [122].

The adoption of One Health strategies highlights the interconnectedness of human, animal, and environmental health in managing zoonotic risks; however, operationalising these approaches at the community level remains challenging, particularly in endemic regions where awareness and resources may be limited. For example, the inconsistent use of protective barriers such as date palm sap covers to prevent bat contamination, along with variable uptake of risk-based farming and horse management practices, such as modifying paddock use, relocating feed and water points, stabling horses during higher-risk periods, and vaccination, underscores the need for scalable, culturally appropriate interventions. Henipaviruses provide a salient illustration of the One Health challenge of mitigating zoonotic disease risk while avoiding deleterious impacts on wildlife populations that perform essential ecological functions. In both HeV and NiV systems, human population growth, land-use change, and agricultural practices appear to have created conditions that facilitate contact between reservoir hosts, intermediate or link species, and humans, underscoring the need for a better understanding of the wild–domestic animal interface and the mechanisms driving zoonotic spillover risk. In the case of HeV, the increasing prevalence of hobby farms and recreational horse ownership in proximity to bat colonies has expanded opportunities for spillover. Similarly, for NiV, the introduction and intensification of date palm sap harvesting have created an ecological interface that attracts bats and promotes contamination of a product consumed by humans. Collectively, these observations raise the possibility that human infection with NiV occurred prior to its formal recognition, with detection only becoming possible after the virus was identified during the initial Malaysian outbreak and the subsequent availability of targeted diagnostic tools.

Henipaviruses illustrate the complexity of implementing biosafety interventions within interconnected human, animal, and environmental systems. Effective prevention strategies must balance zoonotic disease mitigation with wildlife conservation, agricultural sustainability, occupational safety, and community acceptability. Consequently, successful implementation of One Health-oriented biosafety approaches will require coordinated engagement across veterinary, laboratory, wildlife, agricultural, environmental, and public health sectors. While this review is situated within a One Health context, the evidence identified was concentrated predominantly within the human and laboratory domains. Comparatively little evidence was available regarding environmental determinants of exposure risk or their incorporation into biosafety decision-making, highlighting an important area for future One Health research.

### 4.5 Limitations

This review has several limitations. As a structured narrative review, it does not employ formal systematic review or scoping review methodologies and may therefore not have captured all relevant publications or unpublished operational guidance documents. Evidence was primarily derived from English-language literature and publicly accessible documents, potentially introducing language and publication bias. Much of the available experimental evidence relates to NiV rather than HeV, requiring cautious extrapolation between viruses and operational settings. Evidence quality also varied substantially, ranging from controlled experimental studies to observational outbreak reports and expert guidance documents. In addition, many biosafety recommendations remain based on precautionary assumptions because matrix-specific validation data are limited or unavailable.

Furthermore, the literature review alone may not fully capture operational implementation challenges associated with applying WHO LBM4 approaches in clinical, veterinary, and laboratory settings. Future qualitative and mixed-methods studies involving end-users of biosafety guidance may help identify operational barriers, evidence needs, and priority areas for future refinement of WHO LBM4 implementation strategies.

## 5 Conclusion

This review identified several major limitations in the current biosafety evidence base for Hendra and Nipah viruses, including uncertainty surrounding infectious dose thresholds, incomplete understanding of transmission pathways, limited validation of inactivation procedures across relevant matrices, and insufficient operational evidence supporting implementation of risk-based containment approaches in diverse settings.

Strengthening the biosafety evidence base for henipaviruses will directly support implementation of the WHO LBM4 risk-based framework by improving the evidence available for containment decisions, diagnostic workflows, inactivation procedures, and occupational risk management. Given ongoing environmental change, land-use modification, and climate-driven shifts in bat ecology that may increase future spillover opportunities, addressing these evidence gaps remains an urgent One Health priority. More broadly, the approach used in this review may provide a useful model for evaluating the evidence supporting risk-based biosafety guidance for other high-consequence zoonotic pathogens.

## CRediT authorship contribution statement

**S.D. Blacksell:** Writing – review & editing, Writing – original draft, Supervision, Project administration, Funding acquisition, Formal analysis, Conceptualization. **K.K. Le:** Writing – original draft, Data curation. **P.W. Selleck:** Writing – review & editing. **J.R. Young:** Writing – review & editing. **J.T. Paulley:** Writing – review & editing. **G.A. Marsh:** Writing – review & editing, Conceptualization. **M.P. Ward:** Writing – review & editing. **L.J. Gleeson:** Writing – review & editing.

## Funding

This research was funded in part by the 10.13039/100010269Wellcome Trust [grant number 315982/Z/24/Z]. For the purpose of open access, the author has applied a CC BY public copyright licence to any Author Accepted Manuscript version arising from this submission.

## Declaration of competing interest

The authors declare that the research was conducted without any commercial or financial relationships that could be construed as a potential conflict of interest.

## Data Availability

No data was used for the research described in the article.

## Appendix A Supplementary data

Supplementary material: Supplementary tables S1-S3. Detailed biosafety evidence for Hendra virus and Nipah virus, including transmission pathways, infectious dose studies, occupational exposures, and validated disinfection and inactivation methods, with supporting references.

## References

[1] Blacksell S.D., Dhawan S., Kusumoto M., Le KK Summermatter K., O’Keefe J. (2023). The biosafety research road map: the search for evidence to support practices in human and veterinary laboratories. Appl. Biosaf..

[2] World Health Organization (2020). https://www.who.int/publications/i/item/9789240011311.

[3] Eaton B.T., Broder C.C., Middleton D., Wang L.F. (2006). Hendra and Nipah viruses: different and dangerous. Nat. Rev. Microbiol..

[4] Gazal S., Sharma N., Gazal S., Tikoo M., Shikha D., Badroo G.A. (2022). Nipah and Hendra viruses: deadly zoonotic paramyxoviruses with the potential to cause the next pandemic. Pathogens.

[5] Marsh G.A., Wang L.-F. (2012). Hendra and Nipah viruses: why are they so deadly?. Curr. Opin. Virol..

[6] Eby P., Peel A.J., Hoegh A., Madden W., Giles J.R., Hudson P.J. (2023). Pathogen spillover driven by rapid changes in bat ecology. Nature.

[7] Taylor J., Thompson K., Annand E.J., Massey P.D., Bennett J., Eden J.S. (2022). Novel variant Hendra virus genotype 2 infection in a horse in the greater Newcastle region, New South Wales, Australia. One Health..

[8] Centers for Disease Control and Prevention (1999). Outbreak of Hendra-like virus--Malaysia and Singapore, 1998–1999. MMWR Morb. Mortal Wkly. Rep..

[9] Spengler J.R., Lo M.K., Welch S.R., Spiropoulou C.F. (2025). Henipaviruses: epidemiology, ecology, disease, and the development of vaccines and therapeutics. Clin. Microbiol. Rev..

[10] World Health Organization (2025). Nipah Virus Infection - Bangladesh. https://www.who.int/emergencies/disease-outbreak-news/item/2025-DON582.

[11] World Health Organization (2026). Nipah Virus Update: West Bengal, India. https://www.who.int/southeastasia/news/detail/29-01-2026-nipah-virus-updatewb.

[12] Hasebe F., Thuy N.T., Inoue S., Yu F., Kaku Y., Watanabe S. (2012). Serologic evidence of Nipah virus infection in bats, Vietnam. Emerg. Infect. Dis..

[13] Hayman D.T., Suu-Ire R., Breed A.C., JA McEachern, Wang L., Wood J.L. (2008). Evidence of henipavirus infection in west African fruit bats. PLoS One.

[14] Li Y., Wang J., Hickey A.C., Zhang Y., Li Y., Wu Y. (2008). Antibodies to Nipah or Nipah-like viruses in bats, China. Emerg. Infect. Dis..

[15] Wacharapluesadee S., Boongird K., Wanghongsa S., Ratanasetyuth N., Supavonwong P., Saengsen D. (2010). A longitudinal study of the prevalence of Nipah virus in *Pteropus lylei* bats in Thailand: evidence for seasonal preference in disease transmission. Vector Borne Zoonotic Dis..

[16] Harit A.K., Ichhpujani R.L., Gupta S., Gill K.S., Lal S., Ganguly N.K. (2006). Nipah/Hendra virus outbreak in Siliguri, West Bengal, India in 2001. Indian J. Med. Res..

[17] Murray K., Rogers R., Selvey L., Selleck P., Hyatt A., Gould A. (1995). A novel morbillivirus pneumonia of horses and its transmission to humans. Emerg. Infect. Dis..

[18] O’Sullivan J.D., Allworth A.M., Paterson D.L., Snow T.M., Boots R., Gleeson L.J. (1997). Fatal encephalitis due to novel paramyxovirus transmitted from horses. Lancet.

[19] Goh K.J., Tan C.T., Chew N.K., Tan P.S., Kamarulzaman A., Sarji S.A. (2000). Clinical features of Nipah virus encephalitis among pig farmers in Malaysia. N. Engl. J. Med..

[20] Lee K.E., Umapathi T., Tan C.B., Tjia H.T., Chua T.S., Oh H.M. (1999). The neurological manifestations of Nipah virus encephalitis, a novel paramyxovirus. Ann. Neurol..

[21] Paton N.I., Leo Y.S., Zaki S.R., Auchus A.P., Lee K.E., Ling A.E. (1999). Outbreak of Nipah-virus infection among abattoir workers in Singapore. Lancet.

[22] Halpin K., Young P.L., Field H.E., Mackenzie J.S. (2000). Isolation of Hendra virus from pteropid bats: a natural reservoir of Hendra virus. J. Gen. Virol..

[23] Han H.-J., Wen H.-l., Zhou C.-M., Chen F.-F., Luo L.-M., Liu J.-w. (2015). Bats as reservoirs of severe emerging infectious diseases. Virus Res..

[24] Breed A.C., Breed M.F., Meers J., Field H.E. (2011). Evidence of endemic Hendra virus infection in flying-foxes (*Pteropus conspicillatus*)–implications for disease risk management. PLoS One.

[25] Plowright R.K., Field H.E., Smith C., Divljan A., Palmer C., Tabor G. (2008). Reproduction and nutritional stress are risk factors for Hendra virus infection in little red flying foxes (*Pteropus scapulatus*). Proc. Biol. Sci..

[26] Edson D., Peel A.J., Huth L., Mayer D.G., Vidgen M.E., McMichael L. (2019). Time of year, age class and body condition predict Hendra virus infection in Australian black flying foxes (*Pteropus alecto*). Epidemiol. Infect..

[27] Boardman W.S.J., Baker M.L., Boyd V., Crameri G., Peck G.R., Reardon T. (2020). Seroprevalence of three paramyxoviruses; Hendra virus, Tioman virus, cedar virus and a rhabdovirus, Australian bat lyssavirus, in a range expanding fruit bat, the Grey-headed flying fox (*Pteropus poliocephalus*). PLoS One.

[28] Edson D., Field H., McMichael L., Vidgen M., Goldspink L., Broos A. (2015). Routes of Hendra virus excretion in naturally-infected flying-foxes: implications for viral transmission and spillover risk. PLoS One.

[29] Williamson M.M., Hooper P.T., Selleck P.W., Gleeson L.J., Daniels P.W., Westbury H.A. (1998). Transmission studies of Hendra virus (equine morbillivirus) in fruit bats, horses and cats. Aust. Vet. J..

[30] Middleton D.J., Riddell S., Klein R., Arkinstall R., Haining J., Frazer L. (2017). Experimental Hendra virus infection of dogs: virus replication, shedding and potential for transmission. Aust. Vet. J..

[31] van den Hurk S., Yondo A., Velayudhan B.T. (2025). Laboratory diagnosis of Hendra and Nipah: two emerging zoonotic diseases with one health significance. Viruses.

[32] Luby S.P., Rahman M., Hossain M.J., Blum L.S., Husain M.M., Gurley E. (2006). Foodborne transmission of Nipah virus, Bangladesh. Emerg. Infect. Dis..

[33] Hegde S.T., Sazzad H.M.S., Hossain M.J., Alam M.-U., Kenah E., Daszak P. (2016). Investigating rare risk factors for Nipah virus in Bangladesh: 2001–2012. EcoHealth.

[34] Hughes J.M., Wilson M.E., Luby S.P., Gurley E.S., Hossain M.J. (2009). Transmission of human infection with Nipah virus. Clin. Infect. Dis..

[35] Islam M.S., Sazzad H.M., Satter S.M., Sultana S., Hossain M.J., Hasan M. (2016). Nipah virus transmission from bats to humans associated with drinking traditional liquor made from date palm sap, Bangladesh, 2011-2014. Emerg. Infect. Dis..

[36] Salah Uddin Khan M., Hossain J., Gurley E.S., Nahar N., Sultana R., Luby S.P. (2010). Use of infrared camera to understand bats’ access to date palm sap: implications for preventing Nipah virus transmission. EcoHealth.

[37] Gurley E.S., Hegde S.T., Hossain K., Sazzad H.M.S., Hossain M.J., Rahman M. (2017). Convergence of humans, bats, trees, and culture in Nipah virus transmission, Bangladesh. Emerg. Infect. Dis..

[38] Munster V.J., Prescott J.B., Bushmaker T., Long D., Rosenke R., Thomas T. (2012). Rapid Nipah virus entry into the central nervous system of hamsters via the olfactory route. Sci. Rep..

[39] Clayton B.A., Middleton D., Arkinstall R., Frazer L., Wang L.F., Marsh G.A. (2016). The nature of exposure drives transmission of Nipah viruses from Malaysia and Bangladesh in ferrets. PLoS Negl. Trop. Dis..

[40] Mungall B.A., Middleton D., Crameri G., Halpin K., Bingham J., Eaton B.T. (2007). Vertical transmission and fetal replication of Nipah virus in an experimentally infected cat. J. Infect. Dis..

[41] Epstein J.H., Abdul Rahman S., Zambriski J.A., Halpin K., Meehan G., Jamaluddin A.A. (2006). Feral cats and risk for Nipah virus transmission. Emerg. Infect. Dis..

[42] Luby S.P., Gurley E.S., Hossain M.J. (2009). Transmission of human infection with Nipah virus. Clin. Infect. Dis..

[43] Pritchard S., Hornsey E. (2025). The role of infection prevention and control in the mitigation of human-to-human transmission of Nipah virus: a systematic review. Antimicrob. Resist. Infect. Control.

[44] Gurley E.S., Montgomery J.M., Hossain M.J., Islam M.R., Molla M.A., Shamsuzzaman S.M. (2007). Risk of nosocomial transmission of Nipah virus in a Bangladesh hospital. Infect. Control Hosp. Epidemiol..

[45] Mounts A.W., Kaur H., Parashar U.D., Ksiazek T.G., Cannon D.L., Arokiasamy J.T. (2001). A cohort study of health care workers to assess nosocomial transmissibility of Nipah virus, Malaysia, 1999. J. Infect. Dis..

[46] Sazzad H.M., Hossain M.J., Gurley E.S., Ameen K.M., Parveen S., Islam M.S. (2013). Nipah virus infection outbreak with nosocomial and corpse-to-human transmission, Bangladesh. Emerg. Infect. Dis..

[47] Tan C.T., Tan K.S. (2001). Nosocomial transmissibility of Nipah virus. J. Infect. Dis..

[48] Luby S.P. (2013). The pandemic potential of Nipah virus. Antivir. Res..

[49] Eaton B.T., Broder C.C., Wang L.F. (2005). Hendra and Nipah viruses: pathogenesis and therapeutics. Curr. Mol. Med..

[50] Williamson M.M., Hooper P.T., Selleck P.W., Westbury H.A., Slocombe R.F.S. (2001). A Guinea-pig model of Hendra virus encephalitis. J. Comp. Pathol..

[51] Geisbert T.W., Daddario-DiCaprio K.M., Hickey A.C., Smith M.A., Chan Y.P., Wang L.F. (2010). Development of an acute and highly pathogenic nonhuman primate model of Nipah virus infection. PLoS One.

[52] de Wit E., Prescott J., Falzarano D., Bushmaker T., Scott D., Feldmann H. (2014). Foodborne transmission of nipah virus in Syrian hamsters. PLoS Pathog..

[53] Escaffre O., Hill T., Ikegami T., Juelich T.L., Smith T.K., Zhang L. (2018). Experimental infection of Syrian hamsters with aerosolized Nipah virus. J. Infect. Dis..

[54] Mungall B.A., Middleton D., Crameri G., Bingham J., Halpin K., Russell G. (2006). Feline model of acute nipah virus infection and protection with a soluble glycoprotein-based subunit vaccine. J. Virol..

[55] Bossart K.N., Zhu Z., Middleton D., Klippel J., Crameri G., Bingham J. (2009). A neutralizing human monoclonal antibody protects against lethal disease in a new ferret model of acute nipah virus infection. PLoS Pathog..

[56] Edwards S.J., Caruso S., Suen W.W., Jackson S., Rowe B., Marsh G.A. (2021). Evaluation of henipavirus chemical inactivation methods for the safe removal of samples from the high-containment PC4 laboratory. J. Virol. Methods.

[57] AUSVETPLAN (2021). Operational manual, Decontamination Version 5.0. https://animalhealthaustralia.com.au//wp-content/uploads/2023/05/AUSVETPLAN-Operational-manual-Decontamination.pdf.

[58] Smither S.J., Eastaugh L.S., Findlay J.S., O’Brien L.M., Thom R., Lever M.S. (2019). Survival and persistence of Nipah virus in blood and tissue culture media. Emerg. Microbes Infect..

[59] Smither S.J., Eastaugh L.S., O’Brien L.M., Phelps A.L., Lever M.S. (2022). Aerosol survival, disinfection and formalin inactivation of Nipah virus. Viruses.

[60] Huang Y., Xiao S., Song D., Yuan Z. (2022). Evaluation and comparison of three virucidal agents on inactivation of Nipah virus. Sci. Rep..

[61] Imada T., Abdul Rahman M.A., Kashiwazaki Y., Tanimura N., Syed Hassan S., Jamaluddin A. (2004). Production and characterization of monoclonal antibodies against formalin-inactivated Nipah virus isolated from the lungs of a pig. J. Vet. Med. Sci..

[62] Widerspick L., Vázquez C.A., Niemetz L., Heung M., Olal C., Bencsik A. (2022). Inactivation methods for experimental Nipah virus infection. Viruses.

[63] Berhane Y., Berry J.D., Ranadheera C., Marszal P., Nicolas B., Yuan X. (2006). Production and characterization of monoclonal antibodies against binary ethylenimine inactivated Nipah virus. J. Virol. Methods.

[64] Feldmann F., Shupert W.L., Haddock E., Twardoski B., Feldmann H. (2019). Gamma irradiation as an effective method for inactivation of emerging viral pathogens. Am. J. Trop. Med. Hyg..

[65] Pollak N.M., Marsh G.A., Olsson M., McMillan D., Macdonald J. (2023). Rapid, sensitive, and specific, low-resource molecular detection of Hendra virus. One Health..

[66] Watanabe S., Fukushi S., Harada T., Shimojima M., Yoshikawa T., Kurosu T. (2020). Effective inactivation of Nipah virus in serum samples for safe processing in low-containment laboratories. Virol. J..

[67] Eickmann M., Gravemann U., Handke W., Tolksdorf F., Reichenberg S., Müller T.H. (2020). Inactivation of three emerging viruses - severe acute respiratory syndrome coronavirus, Crimean-Congo haemorrhagic fever virus and Nipah virus - in platelet concentrates by ultraviolet C light and in plasma by methylene blue plus visible light. Vox Sang..

[68] Scholte F.E.M., Kabra K.B., Tritsch S.R., Montgomery J.M., Spiropoulou C.F., Mores C.N. (2022). Exploring inactivation of SARS-CoV-2, MERS-CoV, Ebola, Lassa, and Nipah viruses on N95 and KN95 respirator material using photoactivated methylene blue to enable reuse. Am. J. Infect. Control.

[69] World Health Organization (2020). https://www.who.int/publications/i/item/9789240011311.

[70] Lewis C.E., Pickering B. (2022). Livestock and risk group 4 pathogens: researching zoonotic threats to public health and agriculture in maximum containment. ILAR J..

[71] Sinnott T.J., Somboonwit C., Alrabaa S., Shapshak P. (2023). Dangerous risk group-4 (RG-4) emergent viruses. Bioinformation.

[72] Kaku Y., Noguchi A., Marsh G.A., JA McEachern, Okutani A., Hotta K. (2009). A neutralization test for specific detection of Nipah virus antibodies using pseudotyped vesicular stomatitis virus expressing green fluorescent protein. J. Virol. Methods.

[73] Tamin A., Harcourt B.H., Lo M.K., Roth J.A., Wolf M.C., Lee B. (2009). Development of a neutralization assay for Nipah virus using pseudotype particles. J. Virol. Methods.

[74] Nie J., Liu L., Wang Q., Chen R., Ning T., Liu Q. (2019). Nipah pseudovirus system enables evaluation of vaccines in vitro and in vivo using non-BSL-4 facilities. Emerg. Microbes Infect..

[75] Aljofan M., Porotto M., Moscona A., Mungall B.A. (2008). Development and validation of a chemiluminescent immunodetection assay amenable to high throughput screening of antiviral drugs for Nipah and Hendra virus. J. Virol. Methods.

[76] Centers for Disease Control and Prevention (2020). Biosafety in Microbiological and Biomedical Laboratories 6th Edition. https://www.cdc.gov/labs/pdf/SF__19_308133-A_BMBL6_00-BOOK-WEB-final-3.pdf.

[77] Centers for Disease Control and Prevention (2023). Select Agents and Toxins List. https://www.selectagents.gov/sat/list.htm.

[78] Health and Human Services Department (2024). Possession, Use, and Transfer of Select Agents and Toxins; Biennial Review of the List of Select Agents and Toxins. https://www.federalregister.gov/documents/2024/12/17/2024-29583/possession-use-and-transfer-of-select-agents-and-toxins-biennial-review-of-the-list-of-select-agents.

[79] VetVoice (2018). The Story So Far. https://www.vetvoice.com.au/ec/hendra-virus/the-story-so-far/.

[80] Grant W.J. (2023). The Hendra Story: A Triumph for Australian Science. https://diffusion.weblogs.anu.edu.au/2013/07/29/the-hendra-story-a-triumph-for-australian-science/.

[81] Ali R., Mounts A.W., Parashar U.D., Sahani M., Lye M.S., Isa M.M. (2001). Nipah virus among military personnel involved in pig culling during an outbreak of encephalitis in Malaysia, 1998-1999. Emerg. Infect. Dis..

[82] Premalatha G.D., Lye M.S., Ariokasamy J., Parashar U.D., Rahmat R., Lee B.Y. (2000). Assessment of Nipah virus transmission among pork sellers in Seremban, Malaysia. Southeast Asian J. Trop. Med. Public Health.

[83] Chan K.P., Rollin P.E., Ksiazek T.G., Leo Y.S., Goh K.T., Paton N.I. (2002). A survey of Nipah virus infection among various risk groups in Singapore. Epidemiol. Infect..

[84] Gurley E.S., Montgomery J.M., Hossain M.J., Bell M., Azad A.K., Islam M.R. (2007). Person-to-person transmission of Nipah virus in a Bangladeshi community. Emerg. Infect. Dis..

[85] Chua K.B., Lam S.K., Goh K.J., Hooi P.S., Ksiazek T.G., Kamarulzaman A. (2001). The presence of Nipah virus in respiratory secretions and urine of patients during an outbreak of Nipah virus encephalitis in Malaysia. J. Inf. Secur..

[86] Nikolay B., Salje H., Hossain M.J., Khan A., HMS Sazzad, Rahman M. (2019). Transmission of Nipah virus - 14 years of investigations in Bangladesh. N. Engl. J. Med..

[87] Hassan M.Z., Sazzad H.M.S., Luby S.P., Sturm-Ramirez K., Bhuiyan M.U., Rahman M.Z. (2018). Nipah virus contamination of hospital surfaces during outbreaks, Bangladesh, 2013-2014. Emerg. Infect. Dis..

[88] Australian Government Department of Health and Aged Care (2016). Hendra Virus - cDNA National Guidelines for Public Health Units. https://www.health.gov.au/sites/default/files/documents/2020/02/hendra-virus-cdna-national-guidelines-for-public-health-units.docx.

[89] Luby S.P., Hossain M.J., Gurley E.S., Ahmed B.N., Banu S., Khan S.U. (2009). Recurrent zoonotic transmission of Nipah virus into humans, Bangladesh, 2001-2007. Emerg. Infect. Dis..

[90] Queensland Government (2018). Hendra Virus Information for Veterinarians. https://www.business.qld.gov.au/_designs/content/guide-printing2?parent=88234&SQ_DESIGN_NAME=print_layout.

[91] Queensland Government (2014). Hendra Virus Infection Prevention Advice. https://www.health.qld.gov.au/__data/assets/pdf_file/0026/428624/hev-inf-prev-adv.pdf.

[92] NSW Government (2018). Hendra Virus - June 2018 Primefact 970 Eleventh Edition. https://www.dpi.nsw.gov.au/__data/assets/pdf_file/0019/310492/Hendra-Virus-Primefact-970-1.pdf.

[93] Degeling C., Gilbert G.L., Annand E., Taylor M., Walsh M.G., Ward M.P. (2018). Managing the risk of Hendra virus spillover in Australia using ecological approaches: a report on three community juries. PLoS One.

[94] Middleton D., Pallister J., Klein R., Feng Y.R., Haining J., Arkinstall R. (2014). Hendra virus vaccine, a one health approach to protecting horse, human, and environmental health. Emerg. Infect. Dis..

[95] Manyweathers J., Field H., Longnecker N., Agho K., Smith C., Taylor M. (2017). “Why won’t they just vaccinate?” horse owner risk perception and uptake of the Hendra virus vaccine. BMC Vet. Res..

[96] Gouglas D., Thanh Le T., Henderson K., Kaloudis A., Danielsen T., Hammersland N.C. (2018). Estimating the cost of vaccine development against epidemic infectious diseases: a cost minimisation study. Lancet Glob. Health.

[97] Annand E.J., Reid P.A., Johnson J., Gilbert G.L., Taylor M., Walsh M. (2020). Citizens’ juries give verdict on whether private practice veterinarians should attend unvaccinated Hendra virus suspect horses. Aust. Vet. J..

[98] Foo R., Hey Y.Y., Jia Ng J.H., Chionh Y.T., Chia W.N., Kong P.S. (2022). Establishment of a captive cave nectar bat (*Eonycteris spelaea*) breeding Colony in Singapore. J. Am. Assoc. Lab. Anim. Sci..

[99] Wilson S.J., Ward M.P. (2016). Intangible and economic impacts of Hendra virus prevention strategies. Zoonoses Public Health.

[100] (2023). NIPAH Virus Guidelines.

[101] Directorate of Health Services India (2021). NIPAH Virus Infection-Guidelines for Surveillance, Diagnosis, Treatment, Prevention and Control. https://dhs.kerala.gov.in/wp-content/uploads/2021/09/Nipah-Guidelines-9-04-21-2-1.pdf.

[102] World Health Organization (2016). National Guideline for Management, Prevention and Control of Nipah Virus Infection Including Encephalitis. https://www.moh.gov.bt/wp-content/uploads/afd-files/2014/11/WHO-guideline-for-Management-Prevention-and-Control-of-Nipah-Virus-Infection.pdf.

[103] Chattu V.K., Kumar R., Kumary S., Kajal F., David J.K. (2018). Nipah virus epidemic in southern India and emphasizing “One Health” approach to ensure global health security. J. Family Med. Prim. Care.

[104] Donaldson H., Lucey D. (2018). Enhancing preparation for large Nipah outbreaks beyond Bangladesh: preventing a tragedy like Ebola in West Africa. Int. J. Infect. Dis..

[105] Román R.G., Wang L.-F., Lee B., Halpin K., de Wit E., Broder C.C. (2020). Nipah@20: lessons learned from another virus with pandemic potential. mSphere.

[106] Looi L.M., Chua K.B. (2007). Lessons from the Nipah virus outbreak in Malaysia. Malays. J. Pathol..

[107] Nahar N., Asaduzzaman M., Sultana R., Garcia F., Paul R.C., Abedin J. (2017). A large-scale behavior change intervention to prevent Nipah transmission in Bangladesh: components and costs. BMC. Res. Notes.

[108] Khan S.U., Gurley E.S., Hossain M.J., Nahar N., Sharker M.A., Luby S.P. (2012). A randomized controlled trial of interventions to impede date palm sap contamination by bats to prevent nipah virus transmission in Bangladesh. PLoS One.

[109] Halpin K., Graham K., Durr P.A. (2021). Sero-monitoring of horses demonstrates the Equivac(®) HeV Hendra virus vaccine to be highly effective in inducing neutralising antibody titres. Vaccines (Basel).

[110] Broder C.C., Weir D.L., Reid P.A. (2016). Hendra virus and Nipah virus animal vaccines. Vaccine.

[111] Broder C.C., Xu K., Nikolov D.B., Zhu Z., Dimitrov D.S., Middleton D. (2013). A treatment for and vaccine against the deadly Hendra and Nipah viruses. Antivir. Res..

[112] Playford E.G., Munro T., Mahler S.M., Elliott S., Gerometta M., Hoger K.L. (2020). Safety, tolerability, pharmacokinetics, and immunogenicity of a human monoclonal antibody targeting the G glycoprotein of henipaviruses in healthy adults: a first-in-human, randomised, controlled, phase 1 study. Lancet Infect. Dis..

[113] Centers for Disease Control and Prevention (2014). Hendra Virus Disease (HeV). https://www.cdc.gov/vhf/hendra/diagnosis/index.html.

[114] Freiberg A.N., Worthy M.N., Lee B., Holbrook M.R. (2010). Combined chloroquine and ribavirin treatment does not prevent death in a hamster model of Nipah and Hendra virus infection. J. Gen. Virol..

[115] Chong H.T., Kamarulzaman A., Tan C.T., Goh K.J., Thayaparan T., Kunjapan S.R. (2001). Treatment of acute Nipah encephalitis with ribavirin. Ann. Neurol..

[116] Lo M.K., Feldmann F., Gary J.M., Jordan R., Bannister R., Cronin J. (2019). Remdesivir (GS-5734) protects African green monkeys from Nipah virus challenge. Sci. Transl. Med..

[117] White J., Thompson K., van den Berg D., O’Neill G., Mendez D.H., Talwar J. (2025). ‘Pretty devastating’: exploring horse owner and veterinarian lived experiences of the equine Hendra virus. Front. Vet. Sci..

[118] Ching P.K., de los Reyes V.C., Sucaldito M.N., Tayag E., Columna-Vingno A.B., Malbas F.F. (2015). Outbreak of henipavirus infection, Philippines, 2014. Emerg. Infect. Dis..

[119] Siengsanan-Lamont J., Blacksell S.D. (2018). A review of laboratory-acquired infections in the Asia-Pacific: understanding risk and the need for improved biosafety for veterinary and zoonotic diseases. Trop. Med. Infect. Dis..

[120] Siengsanan-Lamont J., Kamolsiripichaiporn S., Ruanchaimun S., Patchimasiri T., Jongrakwattana B., Blacksell S.D. (2019). Biosafety and biosecurity challenges facing veterinary diagnostic Laboratories in Lower-Middle Income Countries in Southeast Asia: a case study of Thailand. Appl Biosaf..

[121] Chua K.B., Chua B.H., Wang C.W. (2002). Anthropogenic deforestation, El Niño and the emergence of Nipah virus in Malaysia. Malays. J. Pathol..

[122] Barker S.C. (2003). The Australian paralysis tick may be the missing link in the transmission of Hendra virus from bats to horses to humans. Med. Hypotheses.

[123] Centers for Disease Control and Prevention (2023). Henipavirus Infections CDC Yellow Book 2024. https://wwwnc.cdc.gov/travel/yellowbook/2024/infections-diseases/henipavirus-infections#clinical.

