# Supplementary material for: Hendra and Nipah viruses: Biosafety evidence for risk-based containment, inactivation practices, and one health preparedness
Source: One Health. 2026 Jun 19;23:101486. doi: 10.1016/j.onehlt.2026.101486 (PMC13316633; doi:10.1016/j.onehlt.2026.101486)
Supplement: Supplementary file 1 — Supplementary material: Supplementary tables S1-S3. Detailed biosafety evidence for Hendra virus and Nipah virus, including transmission pathways, infectious dose studies, occupational exposures, and validated disinfection and inactivation methods, with supporting references. [file mmc1.docx]

Table S1. Detailed pathogen biosafety evidence for Hendra virus. Evidence gap = No indicates validation within the reported experimental conditions only and does not imply generalisability across matrices, virus strains, or operational settings

| Hazards | Hazard identification | Evidence (direct quote) | Reference | Evidence gap? yes/no) |
| --- | --- | --- | --- | --- |
| Modes of Transmission | Contact with secretions of infected animals | “The rate of detection, and the amount of viral RNA, was highest in urine samples (>serum, packed haemocytes >faecal >nasal >oral), identifying urine as the most plausible source of infection for flying-foxes and for horses. Detection in a urine sample was more efficient than detection in urogenital swabs, identifying the former as the preferred diagnostic sample.” | [1] | No |
|  |  | “Horses can be infected by oronasal routes and can excrete HeV in urine and saliva. It is possible to transmit HeV from cats to horses.” | [2] |  |
|  |  | “Beagle dogs were exposed oronasally to Hendra virus/Australia/Horse/2008/Redlands or to blood collected from HeV-infected ferrets…Dogs were reliably infected with HeV and were generally asymptomatic…HeV is not highly pathogenic for dogs, but their oral secretions pose a potential transmission risk to people.” | [3] |  |
| Infectious dose | Guinea pigs | “Recent investigations have shown that guinea pigs are susceptible to low doses of Hendra virus (as low as 50 TCID50) administered via a natural route of infection (intranasally)” | [4] | No |
|  |  | “The dose used (30 000 to 50 000 TCID50), which was higher than in previous studies, produced microscopical lesions of encephalitis in eight of the 15 subcutaneously inoculated guinea-pigs, with positive immunolabelling in blood vessels and neurons, especially in the medulla, cerebellum and thalamus.” | [5] |  |
| Occupationally acquired infections |  | “A veterinarian became infected with Hendra virus (HeV) after managing a terminally ill horse and performing a limited autopsy with inadequate precautions. Although she was initially only mildly ill, serological tests suggested latent HeV infection. Nevertheless, she remains well 2 years after her initial illness.” | [6] | No |

# Table S2. Detailed pathogen biosafety evidence for Nipah virus. Evidence gap = No indicates validation within the reported experimental conditions only and does not imply generalisability across matrices, virus strains, or operational settings

| Hazards | Hazard identification | Evidence (direct quote) | Reference | Evidence gap? (yes/no) |
| --- | --- | --- | --- | --- |
| Modes of Transmission | Consumption of date palm sap | “The only exposure significantly associated with illness was drinking raw date palm sap (64% among case-patients vs. 18% among controls, odds ratio [OR] 7.9 p = 0.01).” | [7] | No |
|  |  | “We conducted a risk factor analysis using all 157 cases and 632 controls surveyed in previous investigations during 2004-2012 to identify exposures independently associated with Nipah, since date palm sap was first asked about as an exposure in 2004. To further explore possible rare exposures, we also conducted in-depth interviews with all cases, or proxies, since 2001 that reported no exposure to date palm sap or contact with another case. Cases were 4.9 (95% 3.2-7.7) times more likely to consume raw date palm sap and 7.3 (95% 4.0-13.4) times more likely to have contact with a Nipah case than controls.” | [8] |  |
|  |  | “The primary pathways of transmission from bats to people in Bangladesh are through contamination of raw date palm sap by bats with subsequent consumption by humans and through infection of domestic animals (cattle, pigs, and goats), presumably from consumption of food contaminated with bat saliva or urine with subsequent transmission to people.” | [9] |  |
|  |  | “A team of epidemiologists and anthropologists investigated these 3 clusters comprising 14 case-patients, 8 of whom died. Among the 14 case-patients, 8 drank fermented date palm sap (tari) regularly before their illness”  “Out of 20 camera-nights of observations, 14 identified 132 visits of bats around the tree, 91 to the shaved surface of the tree where the sap flow originates, 4 at the stream of sap moving toward the collection pot, and no bats at the tap or on the collection pots; the remaining 6 camera-nights recorded no visits…This study confirmed that bats commonly visited date palm trees and physically contacted the sap collected for human consumption.” | [10] |  |
|  |  | “Nipah virus was excreted in bat urine while neutralising antibody was present in serum. This intermittent, low-level excretion of Nipah virus in the urine of bats may be sufficient to sustain the net reproductive value of the virus in a species where there is regular urine contamination of the fur, mutual grooming, and where urine droplets are a feature of the environment.” | [11] |  |
|  | Contact with infected animals | “If fruit bats that are infected with NiV gain access to the sap for drinking, they might occasionally contaminate the sap through saliva and urine. In February 2007, we conducted a qualitative study in six villages, interviewing 27 date palm sap collectors (gachhis) within the geographical area where NiV outbreaks have occurred since 2001. Gachhis reported that bats pose a challenge to successful collection of quality sap, because bats drink and defecate into the sap which markedly reduces its value.” | [12] |  |
|  |  | “Multivariable analyses showed that, compared with nearby control villages, each additional 10% increase in the proportion of households reporting that someone regularly consumed raw sap was associated with a 6.39 (95% CI 1.61–25.40) increase in odds of being a village with cases of NiV infection (Table 2).” | [13] |  |
|  |  | “Here, we performed a spatiotemporal analysis of NiV entry into the CNS of  hamsters. NiV initially predominantly targeted the olfactory epithelium in the nasal turbinates.” | [14] |  |
|  |  | “All naïve ferrets developed acute infection following assisted and direct exposure to oronasal fluid from animals that were shedding either NiV-BD or NiV-MY.” | [15] |  |
|  |  | “A female adult cat developed clinical disease 13 days after subcutaneous inoculation with Nipah virus (NiV) and was discovered to be pregnant at necropsy. Viral genome was detected in a variety of specimens, including blood, serum, tonsil swabs, and urine, up to 3 days before the onset of disease. Samples collected postmortem, including placenta, uterine fluid, and fetal tissues, were also positive for NiV genome, and the placenta and uterine fluid contained high levels of recoverable virus.” | [16] |  |
|  | Human-to-human transmission | “Thirty-six cases of Nipah virus illness were identified; 75% of case-patients died. Multiple peaks of illness occurred, and 33 case-patients had close contact with another Nipah virus patient before their illness. The results from a case‒control study showed that contact with 1 patient carried the highest risk for infection (odds ratio 6.7, 95% confidence interval 2.9–16.8, p<0.001). RT‒PCR testing of environmental samples confirmed Nipah virus contamination of hospital surfaces. This investigation provides evidence for person-to-person transmission of Nipah virus.” | [17] |  |
|  |  | “We identified 16 case-patients; 14 of these patients died. For 1 case-patient, the only known exposure was hugging a deceased patient with a probable case, while another case-patient’s exposure involved preparing the same corpse for burial by removing oral secretions and anogenital excreta with a cloth and bare hands.” | [18] |  |
|  |  | “During the peak outbreak of Nipah virus encephalitis in Malaysia, Nipah virus was isolated from the upper respiratory secretions and urine in eight of 20 patients who were virologically and/or serologically confirmed to be infected with the virus. From these eight patients, Nipah virus was isolated from six throat swab specimens, three urine specimens and only one nasal swab specimen.” | [19] |  |
|  |  | “The risk of infection increased with increased duration of exposure of the contacts (adjusted odds ratio for exposure of >48 hours vs. ≤1 hour, 13; 95% CI, 2.6 to 62) and with exposure to body fluids (adjusted odds ratio, 4.3; 95% CI, 1.6 to 11).” | [20] |  |
| Infectious dose | Syrian hamsters | “Drinking of 5×108 TCID50 of Nipah virus resulted in neurological disease in 5 out of 8 hamsters, indicating that food-borne transmission of Nipah virus can indeed occur. In comparison, intranasal (i.n.) inoculation with the same dose of Nipah virus resulted in lethal respiratory disease in all animals.” | [21] | No |
|  |  | “In an initial experiment, groups of 5 hamsters were exposed via whole-body aerosol to 4 different NiV-M doses ranging from 102 to 105 PFU/animal. Based on the calculated presented doses (Dp) (Figure 1A), the LD50 value was estimated to be lower than 102 PFU in hamsters (Figure 1A).” | [22] |  |
|  | African Green Monkey | “Together, these data indicated that NiV lethality in AGMs occurred within 9 to 12 days after virus infection with an i.t. or i.t and oral exposure with doses ranging from ~2.5x10^3^ pfu to ~1.3x10^6^pfu of NiV” | [23] |  |
|  | Felines | “Each cat exhibited mild to moderately severe histological lesions consistent with that previously reported in NiV infection of cats. These comprised, variously, acute bronchiolitis and focal necrotising alveolitis with syncytium formation in respiratory epithelial cells (Fig. 2A and B), pulmonary arteritis with fibrinoid necrosis of the vessel walls, endothelial syncytial cell formation, and intracytoplasmic eosinophilic inclusion bodies. All cats had focal hemorrhagic and necrotic lesions of the splenic red pulp associated with syncytia. Cat 2 had edema of the serosal, muscular, and submucosal layers of the bladder wall; submucosal hemorrhages with acute inflammatory cell infiltration; and endothelial syncytia. Necrotising lymphadenitis was identified in cat 4. Positive immunostaining for NiV antigen was detected in bronchiolar and alveolar epithelial cells (Fig. 2C and D), in the pulmonary vascular endothelium and tunica media of pulmonary arteries, and within syncytial cells within the lungs and spleens of all four cats. Antigen was also identified in syncytial cells, connective tissue cells, and the endothelium of inflamed bladder submucosa and lymphoid tissue.” | [24] |  |
|  | Ferret | “Here, a new ferret model of Nipah virus pathogenesis is described where both respiratory and neurological disease are present in infected animals. Severe disease occurs with viral doses as low as 500 TCID50 within 6 to 10 days following infection. The underlying pathology seen in the ferret closely resembles that seen in Nipah virus infected humans, characterised as a widespread multisystemic vasculitis, with virus replicating in highly vascular tissues including lung, spleen and brain, with recoverable virus from a variety of tissues.” | [25] |  |
| Occupationally acquired infections |  | “To assess the possibility of nosocomial transmission, 338 health care workers (HCWs) exposed and 288 HCWs unexposed to outbreak-related patients were surveyed, and their serum samples were tested for anti-Nipah virus antibody." | [26] | No |
|  |  | “We conducted a seroprevalence study and exposure survey of healthcare workers to assess the risk of nosocomial transmission of Nipah virus during an outbreak in Bangladesh in 2004. No evidence of recent Nipah virus infection was detected despite substantial exposures and minimal use of personal protective equipment.” | [27] |  |
|  |  | “Between September 1998 and May 1999, 265 cases of encephalitis were reported from among those involved in pig rearing. A few cases were also reported among abattoir workers” | [28] |  |
|  |  | “Our findings indicate that transmissibility of Nipah virus to military personnel involved in pig culling was low. Four of the six infected persons were apparently well; follow-up of these and the other infected soldiers will be important to determine if any symptoms of disease or long-term sequelae develop. The observation that all the infected persons reported direct contact with live pigs is consistent with the hypothesis that transmission of Nipah virus to humans most likely occurs from close contact with infectious secretions or body fluids of pigs.” | [29] |  |
|  |  | “Following the Nipah virus (NV) outbreak in March 1999 in Singapore, a serological survey  was undertaken to screen individuals potentially exposed to NV. Twenty-two (1.5%) of 1469 people tested had antibodies suggesting NV infection. Although 12 of the 22 infected people (54.6%) were  symptomatic, the remaining 10 (45.4%) were clinically well and had no past history of  compatible pulmonary or neurological disease” | [30]. |  |

# Table S3. Detailed Disinfection and inactivation evidence for Hendra and Nipah virus. Evidence gap = No indicates validation within the reported experimental conditions only and does not imply generalisability across matrices, virus strains, or operational settings

| Virus |  | Method | Evidence (direct quote) | Reference | Evidence gap? (yes/no) |
| --- | --- | --- | --- | --- | --- |
| Hendra |  | Chemical | “Henipavirus-infected cell monolayers treated with 4% paraformaldehyde (PFA) showed the complete inactivation of infectious virus, with an inactivation period of 15 min resulting in more than 8-log decrease in infectious titre.” | [31] | Yes |
|  |  |  | “Hendra virus [HeV]…Following irradiation, samples were returned to biocontainment for inactivation testing in tissue culture using an infectivity assay determining the TCID50. Vero cells were infected with a 1:1 (v/v) dilution in DMEM of the irradiated sample. Following a 1-hour adsorption in a small volume (1 mL), an appropriate amount of maintenance medium (DMEM, 2% FBS) was added and cells were monitored daily for cytopathogenic effects over a period of 14 days. Negative cultures were passaged one time in freshly seeded Vero cells and monitored under the same conditions for another 14-day period.” | [32] |  |
|  |  | Radiation | “For inactivation, Nipah virus and Hendra virus stocks were sent to a commercial gamma-irradiator (Steritech, Dandenong, Australia) where they were treated with 50 kGy gamma-irradiation for inactivation.” | [33] |  |
| Nipah |  | Chemical | “…10% NBF to completely inactivate infectious virus within a biological sample was evaluated.” | [31] | No |
|  |  |  | “Sodium hypochlorite (at 10%)…reduced the titre of Nipah virus to undetectable levels…ethanol (at 80%) reduced the titre of Nipah virus to undetectable levels” | [34] |  |
|  |  |  | “19% ethanol was able to inactivate Nipah virus when applied for at least 8 min contact time” | [35] |  |
|  |  |  | “The NV antigens were made safe for handling by inactivation with formalin. NV infected cells in culture flasks were cover with plastic bags, and frozen thawed three times. Formalin was directly added at a final concentration of 0.1%, and the flasks were left for 72 hr at 4oC” | [36] |  |
|  |  |  | “Nipah virus that was in tissue culture medium was also inactivated after 24 h in the presence of 10% formalin” | [34] |  |
|  |  |  | “In our study, we used inactivation methods that involved: two organic solvents (acetone and methanol) and a mixture of both, fluoropolymers/aldehyde fixatives (PFA, C/C and NBF), detergents (SDS and Triton-X 100) and guanidinium isothiocyanate-containing reagents (AVL, RLT and TRIzol). As shown in Figure 4, the inactivation of NiV-infected cells with PFA, C/C and all the inactivation methods involving organic solvents, except for methanol inactivation, presented detectable NiV RNA genome levels that subsequently decreased over the 21-day propagation period (three passages). In contrast, when applying other inactivation methods such as SDS, RLT + EtOH, Triton-X 100 or TRIzol reagent, no detectable NiV RNA genome was observed.” | [37] |  |
|  |  |  | “In this study, the virucidal efficacy of three disinfectants (Micro-Chem Plus detergent disinfectant cleaner, FWD and Medical EtOH) against Nipah virus was evaluated in quantitative suspension tests including. Our results showed that the > 4 log reduction achieved for all products in inactivating Nipah virus in 15 s.” | [35] |  |
|  |  |  | “Complete BEI inactivation of Nipah virus with initial stock virus titre of 2 × 105 TCID50/ml was achieved within 6 h based on virus titration assay from samples taken at different time points. During safety testing CPE was not observed in PT-K75 cells that were propagated with undiluted aliquots of BEI inactivated virus from the final time point (24 h).” | [38] |  |
|  |  | Radiation | “For inactivation, Nipah virus and Hendra virus stocks were sent to a commercial gamma-irradiator (Steritech, Dandenong Australia) where they were treated with 50 kGy gamma-irradiation for inactivation.” | [33] | No |
|  |  |  | “Nipah virus [NiV]…Following irradiation, samples were returned to biocontainment for inactivation testing in tissue culture using an infectivity assay determining the TCID50. Vero cells were infected with a 1:1 (v/v) dilution in DMEM of the irradiated sample. Following a 1-hour adsorption in a small volume (1 mL), an appropriate amount of maintenance medium (DMEM, 2% FBS) was added and cells were monitored daily for cytopathogenic effects over a period of 14 days. Negative cultures were passaged one time in freshly seeded Vero cells and monitored under the same conditions for another 14-day period.” | [32] |  |
|  |  |  | “For UV light inactivation, 5 mL of supernatant was put in a Petri dish and, with the lid open, was exposed to UV light (Sankyo Denki G30T8 Germicidal Lamp, 30 W, AKA 254 nm) inside a Class II laminar hood for 1 h. The distance from the UV lamp was approximately 10 cm” | [37] |  |
|  |  |  | “Furthermore, sample tubes placed on the transilluminator were all covered with aluminum foil (Fig. 1) to reflect the transmitted and scattered UV lights to the target sample [15]. Whereas UV irradiation for 10 min effectively inactivated the infectious NiV spiked in the growth medium (Fig. 3b), the virus in pooled human serum was not eliminated by the 10-min irradiation (more than 1 × 104 TCID50/mL of NiV remained inactivated) (Fig. 3a). This result suggests that excess serum proteins prevent the virus from being UV-irradiated. However, 30-min UV irradiation of the pooled human serum spiked with infectious NiV was effective for virus inactivation (Fig. 3a).” | [39] |  |
|  |  | Combination of chemicals and light | “Treatment with half to three‐fourths of the full UVC dose (0·2 J/cm2) reduced the infectivity of … NiV (≥4·3 log) to the limit of detection (LOD) in platelet concentrates, and treatment with MB and a fourth of the full light dose (120 J/cm2) decreased that of … NiV (≥2·7 log) to the LOD in plasma.” | [40] | No |
|  |  |  | “Virus-MB mixtures were placed in empty tissue culture plates and exposed to bright white light (50,000 lux) for 30 min…whereas NiV needed 10 µM MB and bright light for complete inactivation (Fig 1)." | [41] |  |
|  |  | Heat | “Because certain RNA viruses are not necessarily inactivated by heating at 56 °C for 30 min, the inactivation efficacy of the heating and UV-radiation conditions (56 °C for 30 or 60 min and 60 °C for 30 min) was tested to evaluate the thermal stability of NiV. The viral titres of NiV spiked in pooled human serum before and after the heat treatment were determined (Fig. 2). After heating at 56 °C for 30 min, the NiV titre (1.04 × 107 TCID50/mL) decreased by more than 4 log10. In the triplicate test, the titre decreased to the detection limit (1.39 × 102 TCID50/mL) twice, whereas the titre decreased below the detection limit once (< 1.39 × 102 TCID50/mL) (Fig. 2). The treatments at 56 °C for 60 min and with 60 °C for 30 min resulted in NiV infectious titres below the detection limit in all triplicate tests (Fig. 2 and data not shown).” | [39] | No |

# References

1. Edson D, Field H, McMichael L, Vidgen M, Goldspink L, Broos A, et al. Routes of Hendra Virus Excretion in Naturally-Infected Flying-Foxes: Implications for Viral Transmission and Spillover Risk. PLoS One. 2015;10(10):e0140670.

2. Williamson MM, Hooper PT, Selleck PW, Gleeson LJ, Daniels PW, Westbury HA, et al. Transmission studies of Hendra virus (equine morbillivirus) in fruit bats, horses and cats. Aust Vet J. 1998;76(12):813-8.

3. Middleton DJ, Riddell S, Klein R, Arkinstall R, Haining J, Frazer L, et al. Experimental Hendra virus infection of dogs: virus replication, shedding and potential for transmission. Aust Vet J. 2017;95(1-2):10-8.

4. Eaton BT, Broder CC, Wang LF. Hendra and Nipah viruses: pathogenesis and therapeutics. Curr Mol Med. 2005;5(8):805-16.

5. Williamson MM, Hooper PT, Selleck PW, Westbury HA, Slocombe RFS. A Guinea-pig Model of Hendra Virus Encephalitis. Journal of Comparative Pathology. 2001;124(4):273-9.

6. Hanna JN, McBride WJ, Brookes DL, Shield J, Taylor CT, Smith IL, et al. Hendra virus infection in a veterinarian. Med J Aust. 2006;185(10):562-4.

7. Luby SP, Rahman M, Hossain MJ, Blum LS, Husain MM, Gurley E, et al. Foodborne transmission of Nipah virus, Bangladesh. Emerg Infect Dis. 2006;12(12):1888-94.

8. Hegde ST, Sazzad HMS, Hossain MJ, Alam M-U, Kenah E, Daszak P, et al. Investigating Rare Risk Factors for Nipah Virus in Bangladesh: 2001–2012. EcoHealth. 2016;13(4):720-8.

9. Hughes JM, Wilson ME, Luby SP, Gurley ES, Hossain MJ. Transmission of Human Infection with Nipah Virus. Clinical Infectious Diseases. 2009;49(11):1743-8.

10. Islam MS, Sazzad HM, Satter SM, Sultana S, Hossain MJ, Hasan M, et al. Nipah Virus Transmission from Bats to Humans Associated with Drinking Traditional Liquor Made from Date Palm Sap, Bangladesh, 2011-2014. Emerg Infect Dis. 2016;22(4):664-70.

11. Middleton DJ, Morrissy CJ, van der Heide BM, Russell GM, Braun MA, Westbury HA, et al. Experimental Nipah Virus Infection in Pteropid Bats (Pteropus poliocephalus). Journal of Comparative Pathology. 2007;136(4):266-72.

12. Nahar N, Sultana R, Gurley ES, Hossain MJ, Luby SP. Date Palm Sap Collection: Exploring Opportunities to Prevent Nipah Transmission. EcoHealth. 2010;7(2):196-203.

13. Gurley ES, Hegde ST, Hossain K, Sazzad HMS, Hossain MJ, Rahman M, et al. Convergence of Humans, Bats, Trees, and Culture in Nipah Virus Transmission, Bangladesh. Emerg Infect Dis. 2017;23(9):1446-53.

14. Munster VJ, Prescott JB, Bushmaker T, Long D, Rosenke R, Thomas T, et al. Rapid Nipah virus entry into the central nervous system of hamsters via the olfactory route. Scientific Reports. 2012;2(1):736.

15. Clayton BA, Middleton D, Arkinstall R, Frazer L, Wang LF, Marsh GA. The Nature of Exposure Drives Transmission of Nipah Viruses from Malaysia and Bangladesh in Ferrets. PLoS Negl Trop Dis. 2016;10(6):e0004775.

16. Mungall BA, Middleton D, Crameri G, Halpin K, Bingham J, Eaton BT, et al. Vertical transmission and fetal replication of Nipah virus in an experimentally infected cat. J Infect Dis. 2007;196(6):812-6.

17. Gurley ES, Montgomery JM, Hossain MJ, Bell M, Azad AK, Islam MR, et al. Person-to-person transmission of Nipah virus in a Bangladeshi community. Emerg Infect Dis. 2007;13(7):1031-7.

18. Sazzad HM, Hossain MJ, Gurley ES, Ameen KM, Parveen S, Islam MS, et al. Nipah virus infection outbreak with nosocomial and corpse-to-human transmission, Bangladesh. Emerg Infect Dis. 2013;19(2):210-7.

19. Chua KB, Lam SK, Goh KJ, Hooi PS, Ksiazek TG, Kamarulzaman A, et al. The Presence of Nipah Virus in Respiratory Secretions and Urine of Patients during an Outbreak of Nipah Virus Encephalitis in Malaysia. Journal of Infection. 2001;42(1):40-3.

20. Nikolay B, Salje H, Hossain MJ, Khan A, Sazzad HMS, Rahman M, et al. Transmission of Nipah Virus - 14 Years of Investigations in Bangladesh. N Engl J Med. 2019;380(19):1804-14.

21. de Wit E, Prescott J, Falzarano D, Bushmaker T, Scott D, Feldmann H, et al. Foodborne transmission of nipah virus in Syrian hamsters. PLoS Pathog. 2014;10(3):e1004001.

22. Escaffre O, Hill T, Ikegami T, Juelich TL, Smith TK, Zhang L, et al. Experimental Infection of Syrian Hamsters With Aerosolized Nipah Virus. The Journal of Infectious Diseases. 2018;218(10):1602-10.

23. Geisbert TW, Daddario-DiCaprio KM, Hickey AC, Smith MA, Chan YP, Wang LF, et al. Development of an acute and highly pathogenic nonhuman primate model of Nipah virus infection. PLoS One. 2010;5(5):e10690.

24. Mungall BA, Middleton D, Crameri G, Bingham J, Halpin K, Russell G, et al. Feline model of acute nipah virus infection and protection with a soluble glycoprotein-based subunit vaccine. J Virol. 2006;80(24):12293-302.

25. Bossart KN, Zhu Z, Middleton D, Klippel J, Crameri G, Bingham J, et al. A neutralizing human monoclonal antibody protects against lethal disease in a new ferret model of acute nipah virus infection. PLoS Pathog. 2009;5(10):e1000642.

26. Mounts AW, Kaur H, Parashar UD, Ksiazek TG, Cannon DL, Arokiasamy JT, et al. A Cohort Study of Health Care Workers to Assess Nosocomial Transmissibility of Nipah Virus, Malaysia, 1999. The Journal of Infectious Diseases. 2001;183(5):810-3.

27. Gurley ES, Montgomery JM, Hossain MJ, Islam MR, Molla MA, Shamsuzzaman SM, et al. Risk of nosocomial transmission of Nipah virus in a Bangladesh hospital. Infect Control Hosp Epidemiol. 2007;28(6):740-2.

28. Premalatha GD, Lye MS, Ariokasamy J, Parashar UD, Rahmat R, Lee BY, et al. Assessment of Nipah virus transmission among pork sellers in Seremban, Malaysia. Southeast Asian J Trop Med Public Health. 2000;31(2):307-9.

29. Ali R, Mounts AW, Parashar UD, Sahani M, Lye MS, Isa MM, et al. Nipah virus among military personnel involved in pig culling during an outbreak of encephalitis in Malaysia, 1998-1999. Emerg Infect Dis. 2001;7(4):759-61.

30. Chan KP, Rollin PE, Ksiazek TG, Leo YS, Goh KT, Paton NI, et al. A survey of Nipah virus infection among various risk groups in Singapore. Epidemiol Infect. 2002;128(1):93-8.

31. Edwards SJ, Caruso S, Suen WW, Jackson S, Rowe B, Marsh GA. Evaluation of henipavirus chemical inactivation methods for the safe removal of samples from the high-containment PC4 laboratory. Journal of Virological Methods. 2021;298:114287.

32. Feldmann F, Shupert WL, Haddock E, Twardoski B, Feldmann H. Gamma Irradiation as an Effective Method for Inactivation of Emerging Viral Pathogens. Am J Trop Med Hyg. 2019;100(5):1275-7.

33. Pollak NM, Marsh GA, Olsson M, McMillan D, Macdonald J. Rapid, sensitive, and specific, low-resource molecular detection of Hendra virus. One Health. 2023;16:100504.

34. Smither SJ, Eastaugh LS, O'Brien LM, Phelps AL, Lever MS. Aerosol Survival, Disinfection and Formalin Inactivation of Nipah Virus. Viruses. 2022;14(9).

35. Huang Y, Xiao S, Song D, Yuan Z. Evaluation and comparison of three virucidal agents on inactivation of Nipah virus. Scientific Reports. 2022;12(1):11365.

36. Imada T, Abdul Rahman MA, Kashiwazaki Y, Tanimura N, Syed Hassan S, Jamaluddin A. Production and characterization of monoclonal antibodies against formalin-inactivated Nipah virus isolated from the lungs of a pig. J Vet Med Sci. 2004;66(1):81-3.

37. Widerspick L, Vázquez CA, Niemetz L, Heung M, Olal C, Bencsik A, et al. Inactivation Methods for Experimental Nipah Virus Infection. Viruses. 2022;14(5).

38. Berhane Y, Berry JD, Ranadheera C, Marszal P, Nicolas B, Yuan X, et al. Production and characterization of monoclonal antibodies against binary ethylenimine inactivated Nipah virus. Journal of Virological Methods. 2006;132(1):59-68.

39. Watanabe S, Fukushi S, Harada T, Shimojima M, Yoshikawa T, Kurosu T, et al. Effective inactivation of Nipah virus in serum samples for safe processing in low-containment laboratories. Virology Journal. 2020;17(1):151.

40. Eickmann M, Gravemann U, Handke W, Tolksdorf F, Reichenberg S, Müller TH, et al. Inactivation of three emerging viruses - severe acute respiratory syndrome coronavirus, Crimean-Congo haemorrhagic fever virus and Nipah virus - in platelet concentrates by ultraviolet C light and in plasma by methylene blue plus visible light. Vox Sang. 2020;115(3):146-51.

41. Scholte FEM, Kabra KB, Tritsch SR, Montgomery JM, Spiropoulou CF, Mores CN, et al. Exploring inactivation of SARS-CoV-2, MERS-CoV, Ebola, Lassa, and Nipah viruses on N95 and KN95 respirator material using photoactivated methylene blue to enable reuse. American Journal of Infection Control. 2022;50(8):863-70.
